# Data-driven insights into the performance of scalable magnetic clay-based composites for pollutant removal

**DOI:** 10.1039/d6ra01195k

**Published:** 2026-05-19

**Authors:** Maurizio Vespignani, Simona Ortelli, Magda Blosi, Ilaria Zanoni, Milad Takhsha, Franca Albertini, Irini Furxhi, Marina Naldi, Wendy Mensah, Alice Piraccini, Anna Luisa Costa

**Affiliations:** a CNR-ISSMC, National Research Council of Italy-Institute of Science, Technology and Sustainability for Ceramics Via Granarolo 64 48018 Faenza Italy simona.ortelli@issmc.cnr.it magda.blosi@issmc.cnr.it; b Department of Chemical Science, Life and Environmental Sustainability, Parma University Parco Area Delle Scienze, 11A 43124 Parma Italy; c CNR-IMEM, National Research Council of Italy-Institute of Materials for Electronics and Magnetism Parco Area Delle Scienze, 37/A 43124 Parma Italy; d Department of Pharmacy and Biotechnology, Alma Mater Studiorum University of Bologna Via Belmeloro, 6 40126 Bologna Italy

## Abstract

Magnetic clay-based composites are promising materials for pollutant remediation due to their tunable surface chemistry, ion-exchange capacity and facile magnetic recovery. However, their practical deployment remains limited by poor scalability and the lack of a predictive framework linking physicochemical properties to adsorption performance. Here, we address both challenges by combining scalable spray-drying fabrication with colloidally driven design and data-driven modelling, establishing a predictive approach to multifunctional adsorption. We developed a scalable strategy to integrate Fe_3_O_4_ nanoparticles into oppositely charged clay matrices *via* heterocoagulation or coprecipitation, followed by one-step spray-drying to produce mechanically robust micrometre-sized granules. The resulting composites retained the intrinsic magnetic behaviour of Fe_3_O_4_ (M–H up to 1.5 T) while exhibiting strong and selective uptake across multiple pollutant classes, including heavy-metal ions (Cu^2+^ and Fe^3+^ > 15 mg g^−1^), oppositely charged dyes (rhodamine B ∼20 mg g^−1^; methylene blue and methyl orange ∼7 mg g^−1^) and phenols (30–40 µg g^−1^). The co-aggregates were highly reusable, with < 10% efficiency loss after four reuse cycles. Notably, in all cases, chemisorption governed the overall uptake, primarily driven by ion-exchange interactions with aqueous species. A machine-learning model (*R*^2^ = 0.91) correlated adsorption capacity with material descriptors, identifying zeta potential, isoelectric point and hydrodynamic size as the dominant factors controlling performance. Together, these results moved beyond empirical material design by providing a scalable and predictive structure–property framework for magnetic clay composites, enabling their rational optimisation and practical implementation in water treatment.

## Abstract 2

Magnetic clay-based composites are promising materials for pollutant remediation due to their tunable surface chemistry, ion-exchange capacity and facile magnetic recovery.

## Graphical abstract

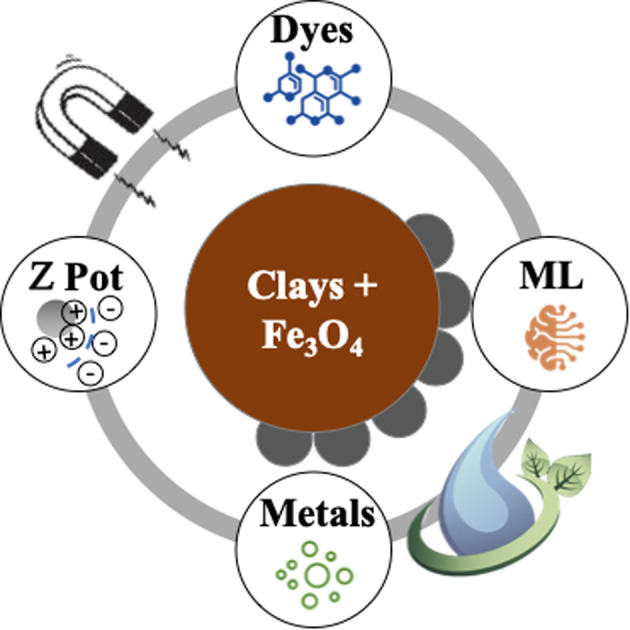

## 1 Introduction

Freshwater availability continues to decline due to the combined effects of climate change, industrialisation and population growth. In contrast, only a limited fraction of global freshwater resources is directly accessible for human use. This scarcity is particularly severe in low-income regions, where water contamination remains a major cause of disease and socio-economic instability.^1^ To address the growing impact of anthropogenic pollutants, several treatment technologies, including coagulation–flocculation, biodegradation, adsorption, membrane separation and advanced oxidation, are routinely applied in industrial and municipal settings.^2–9^ However, these technologies often suffer from high operational costs, energy demand and limited scalability, highlighting the need for more efficient and adaptable treatment materials.^10^ Nanomaterials have emerged as an important class of water-remediation agents due to their high surface activity, tunability and ability to target contaminants with improved selectivity.^11–14^ Among them, iron oxide-based magnetic nanoparticles (MNPs) are particularly attractive owing to their high adsorption capacity and facile recovery under external magnetic fields.^15–18^ Embedding MNPs within stabilising matrices, such as biochars, polymers, mesoporous oxides, zeolites and clay minerals, has therefore emerged as a promising strategy to enhance adsorptive performance and reusability while reducing nanosafety concerns.^19–22^ Despite these advancements, several challenges remain, including the need for scalable and cost-efficient production routes, improved selectivity toward specific pollutants in complex matrices, enhanced long-term stability and reusability of the materials, and the development of greener, more sustainable synthesis strategies, all of which represent key directions for future research.^23^ Clay minerals are particularly appealing as support matrices due to their natural abundance, low cost, structural robustness and favourable surface chemistry, including a high specific surface area, ion-exchange capacity and well-defined surface charge. Numerous studies have reported clay-magnetite (Fe_3_O_4_) composites with a strong ability to remove metal ions (*e.g.*, Cu^2+^, Zn^2+^, Cr^6+^) and organic dyes.^9,24–27^ However, most of the synthetic routes developed to date remain laboratory-scale, multistep and difficult to scale up, limiting the practical deployment of these materials in continuous or industrial processes.^28,29^ Spray drying represents a compelling alternative to conventional synthesis routes because it enables the rapid, continuous and scalable conversion of colloidal suspensions into dry, flowable microparticles with controlled composition and morphology. Through a one-step process, composites with an almost spherical morphology can be produced, with a compositional gradient reflecting that established at the colloidal state, before atomisation.^21,30–32^ Despite its widespread application in the food and pharmaceutical industries, spray drying remains largely underutilised for the fabrication of multiphase inorganic structures, such as magnetic clay-based composites for water purification.^33,34^ To overcome the limitations of conventional batch methods, we propose a strategy that integrates colloidal assembly (*via* heterocoagulation or coprecipitation) with one-step spray drying. This combination provides a streamlined, scalable alternative to traditional multi-step routes, which typically rely on coprecipitation followed by energy-intensive drying stages.^35,36^ However, scalable synthesis alone is not sufficient to guide the rational development of efficient adsorbents, because performance in multi-component clay-Fe_3_O_4_ systems is governed by the combined and often non-linear effects of composition, surface charge, particle size, pH-dependent behaviour, contact time, and pollutant type. Therefore, a central objective of this study was also to establish a data-driven approach to link their physicochemical descriptors to pollutant-removal performance. In this context, machine learning (ML) algorithms, such as artificial neural networks (ANN) and random forest (RF), can capture complex structure–property-performance relationships and help identify the material descriptors that most strongly influence adsorption capacity.^37–40^ Accordingly, in this work, we systematically evaluated how different synthesis routes affect the physicochemical, magnetic, and adsorption properties of clay-Fe_3_O_4_ composites and used ML to correlate adsorption capacity with key material descriptors. The predictive model was complemented by feature-importance analyses (including SHAP and permutation-based methods) to identify the dominant factors controlling pollutant uptake. By combining scalable spray-drying fabrication with data-driven interpretation, this study provides both a practical production route and a rational design for next-generation magnetic adsorbents tailored to different classes of water contaminants.

## 2 Materials and methods

### 2.1 Materials

Several clays were selected based on their crystallographic structure, surface charge, ionic exchange capacity, and specific surface area. Sodium bentonite (NaB) and calcium bentonite (CaB) were purchased by Laviosa Chimica Mineraria (Livorno, Italy), hydrotalcite (LDH2) was purchased by Sasol (Sandton, South Africa) and montmorillonite (MMT) was purchased from the Clay Mineral Society (Chantilly, Virginia, USA). Sepiolite (Sep), Montmorillonite K10 (MMTK10), magnetite (Fe_3_O_4_), iron chloride(ii) tetrahydrate, iron chloride(iii) hexahydrate, copper chloride (CuCl_2_), rhodamine B (RhB), methyl orange (MO), methylene blue (MB) and phenols were purchased from Merck KGaA (Darmstadt, Germany). All the reagents were used as received without further purification.

### 2.2 Methods

**Fig. 1:**
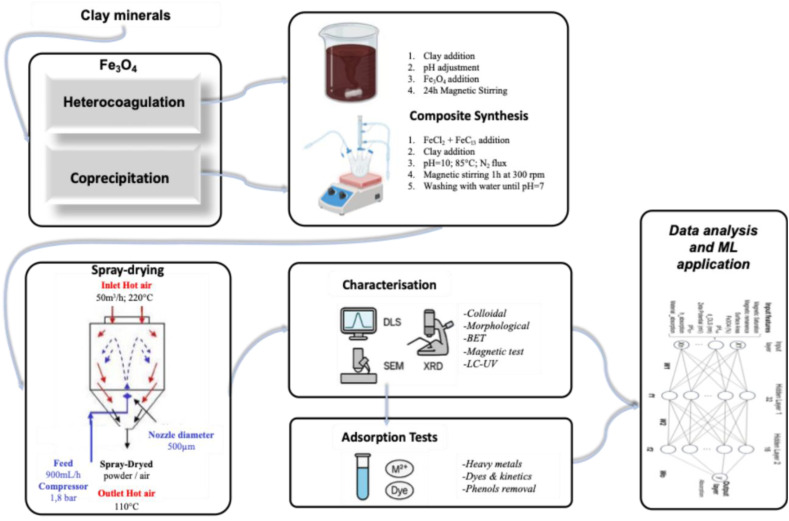
Step-by-step approach for the design and evaluation of magnetic clay-based composites. Steps can be summarised in: (1) composite synthesis, (2) physicochemical and functional characterisation, and (3) data analysis through ML exploitation.

This study employed a stepwise approach to synthesise and characterise magnetic clay-based composites. The process begins with the preparation of Clay-Fe_3_O_4_ composites *via* two routes: heterocoagulation and nucleation. These suspensions are then subjected to spray drying to yield micro-sized, nanostructured powders. The resulting materials are characterised through physicochemical techniques, followed by functional testing for heavy metal, dye, and phenol adsorption in aqueous media and a final analysis through ML. A scheme of the overall process is provided in Fig. 1.

#### 2.2.1 Synthesis by heterocoagulation (Clay/Fe_3_O_4__HC)

Fe_3_O_4_ powder was mixed with aqueous clay suspensions, and the pH was adjusted to a range where the material surfaces carried opposite charges to enhance electrostatic interactions (Table S1). We prepared heterocoagulated samples (HC) by testing two Clay : Fe_3_O_4_ weight ratios (1 : 1 and 5 : 1) and keeping them under magnetic stirring for 24 h.

#### 2.2.2 Synthesis by coprecipitation (Clay@Fe_3_O_4__CP)

Composites were synthesised by following the iron oxides coprecipitation method, as already reported in the literature.^41^ Briefly, a solution of iron(ii) and iron(iii) was prepared by dissolving 1 g of FeCl_2_·4H_2_O and 2.5 g of FeCl_3_·6H_2_O in 150 ml of distilled water (1 : 2 molar ratio). Subsequently, 3 g of clay powder was added to the prepared solution and magnetically stirred at 300 rpm for 10 min to promote clay and ions intercalation. Later, the solution was subjected to precipitation by adding 1 M NaOH solution drop by drop until a black precipitate was observed (pH = 10). The reaction was carried out at 85 °C under N_2_ flux for a reaction time of 1 h. The resulting solution was allowed to settle down at room temperature and then was washed several times with distilled water until pH = 7 to remove unfixed iron oxide compounds. The washing process was performed through centrifugation at 4500 rpm for 10 min. We prepared coprecipitated samples (CP) at a Clay : Fe_3_O_4_ weight ratio of 3 : 1.

#### 2.2.3 Spray drying

The heterocoagulated and coprecipitated samples were dispersed in water (5 wt%) and subsequently spray-dried to obtain free-flowing, easy-to-handle powders. Spray drying was performed using a lab-scale SD-06 spray dryer (LabPlant, North Yorkshire, UK) with hot air as the drying medium. The operating conditions are summarised in Fig. 1: an inlet air flow of 50 m^3^ h^−1^ at 220 °C, an outlet temperature of 110 °C, and a feed rate of 900 mL h^−1^ at 1.8 bar.

#### 2.2.4 Physicochemical characterisation

##### 2.2.4.1 Dynamic and electrophoretic light scattering

We measured the colloidal properties of as-synthesised powders dispersed in water at 0.1 g L^−1^, before spray-drying. Particle size distribution and zeta potential were determined at 25 °C by using a Zetasizer Nanoseries (Malvern Instruments, Malvern, UK) *via* Dynamic Light Scattering (DLS) and Electrophoretic Light Scattering (ELS) techniques, respectively. The Smoluchowski equation was applied to convert the electrophoretic mobility to zeta potential. After a 2 min temperature equilibration step, samples underwent three measurements; the hydrodynamic diameter and zeta potential were obtained by averaging these measurements. The instrument is equipped with an auto-titration unit, which enables exploring the zeta-potential trend within a selected pH range and identifies the isoelectric point (pH_IEP_), corresponding to the pH at which zeta potential is neutralised.

##### 2.2.4.2 Scanning electron microscopy

The powder morphology was investigated by Field Emission Gun-Scanning Electron Microscope (FEG-SEM, Carl Zeiss Microscopy GmbH, Germany) operating at 5–10 keV, equipped with a dispersion micro-analysis of energy (EDS) (INCA, Oxford Instruments, UK) and the scanning transmission electron microscopy (STEM) accessory (Carl Zeiss Microscopy GmbH, Germany). To collect SEM images, samples were placed on carbon films and coated with gold using the coating apparatus Quorum Q 150T ES (Quorum Technologies Ltd, Laughton, UK) to create electric conductivity.

##### 2.2.4.3 X-ray diffraction

X-ray diffraction spectra of samples at room temperature were collected with a Bragg/Brentano diffractometer (X'pertPro Panalytical, Malvern Panalytical, Malvern, UK) equipped with a fast X'Celerator detector, using a Cu anode as the X-ray source (Kα, *λ* = 0.15418 nm). Diffractograms were recorded in the range 5–80° 2*θ*, counting for 0.5 s every 0.02° 2*θ* step.

##### 2.2.4.4 Specific surface area by the BET method

The specific surface areas were measured by N_2_ physisorption apparatus (Surfer, Thermo Fisher Scientific, Waltham, Massachusetts, USA) *via* Brunauer–Emmett–Teller (BET) analysis, in which samples were pre-treated under a vacuum (*P* < 4 mbar) at 200 °C for 1 h.

##### 2.2.4.5 Magnetic test

The magnetisation as a function of the external magnetic field, at room temperature, was measured by a vibrating sample magnetometer (VSM) model 7400 from Lake Shore (Westerville, Ohio, USA), applying a magnetic field range of *µ*_0_H = ±1.5 T. Around 30 mg of the samples were compressed into the sample-holder and analysed. Magnetisation was expressed as emu per g_Fe_3_O_4__^−1^ (same as Am^2^ Kg_Fe_3_O_4__^−1^) present in the composite based on ICP-OES analysis.

#### 2.2.5 Functional characterisation

##### 2.2.5.1 Equilibrium adsorption capacity

The equilibrium adsorption capacity (*q*_e_) of the clay-based composites was calculated using the following mass-balance equation (eqn (1)):1
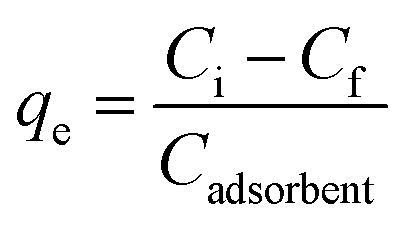
where *C*_i_ and *C*_f_ are the initial and equilibrium concentrations of the aqueous pollutants, respectively, and *C*_adsorbent_ is the concentration of other adsorbent in the suspension (g L^−1^). For heavy metals and dyes, *q*_e_ is expressed in mg g^−1^, while for phenols it is reported in µg g^−1^ due to the lower concentration ranges investigated.

##### 2.2.5.2 Heavy metal adsorption test

Batch adsorption experiments were conducted using CuCl_2_ (100 mg L^−1^) and FeCl_3_·6H_2_O (75 mg L^−1^) simulant solutions (100 ml). The tests were performed at 25 °C under magnetic stirring (500 rpm). Based on optimised literature conditions, the working pH was set at 4.5 for Cu^2+^ and 3.5 for Fe^3+^, with adsorbent dosages of 2.5 g L^−1^ and 1 g L^−1^, respectively. After contact times of 1 h and 24 h, the mixtures were ultrafiltered (Amicon filters Polyethersulfone 3 KDa, Millipore) and centrifuged (4500 rpm for 40 min). The filtrate was analysed *via* ICP-OES (Agilent 5100) in radial viewing mode. All reported data are the result of instrumental triplicate measurements.

##### 2.2.5.3 Dye adsorption test and kinetic studies

Adsorption of Rhodamine B (RhB, 7 mg L^−1^), Methyl Orange (MO, 20 mg L^−1^), and Methylene Blue (MB, 7 mg L^−1^) was performed in 100 ml solutions at pH = 7 and 25 °C, using an adsorbent dose of 1 g L^−1^. For kinetic studies, samples were collected at intervals of 5, 10, 20, 30, 45, and 60 minutes. Aliquots were centrifuged (4500 rpm, 15 min) and analysed using a UV-vis spectrophotometer (Hach Lange DR3900) at the respective maximum absorption wavelengths (553 nm for RhB, 464 nm for MO, and 664 nm for MB). Data were fitted using pseudo-first-order and pseudo-second-order kinetic models.

##### 2.2.5.4 Phenols removal test

Phenol removal experiments were carried out at 25 °C. A multi-component solution was prepared containing six phenolic compounds (phenol, 4-nitrophenol, 2-chlorophenol, 2-nitrophenol, 4-chloro-3-methylphenol, and 2,4-dichlorophenol) at a total concentration of 12 mg L^−1^ (2.0 mg L^−1^ each). The solution was incubated with an adsorbent dosage of 40 g L^−1^ of adsorbent under continuous stirring. Aliquots were collected at defined time intervals (from 0.17 to 72 h), centrifuged, and the supernatant was analysed using a Jasco HPLC system equipped with a C18 column (150 × 4.6 mm, 5 µm, 1000 nm; Phenomenex Kinetex) and a UV-vis detector set at 280 nm. Separation was achieved *via* gradient elution using water and acetonitrile, both acidified with 0.04%v/v trifluoroacetic acid. All analyses were performed in duplicate for each independent sample.

##### 2.2.5.5 Reusability studies

Reusability tests were conducted to evaluate the stability and economic feasibility of the best-performing co-aggregates, using both Cu^2+^ and dye removal as representative model systems. The experiments were standardised under the following conditions: an initial metal concentration of 10 mg L^−1^, dye concentrations of 30 mg L^−1^, an adsorbent dosage of 1 g L^−1^, pH = 3 for Cu^2+^ and pH = 7 for dyes, and a contact time of 120 min at 25 °C. After each adsorption cycle, the material was recovered by magnetic separation and reused in the subsequent run. A total of four consecutive cycles was carried out to assess the retention of removal efficiency over repeated use.

##### 2.2.5.6 Statistical analysis

All adsorption experiments were performed at least in duplicate or triplicate, as specified in the corresponding experimental sections. Results are reported as mean values, and standard deviations were calculated where replicate measurements were available. For heavy-metal adsorption, ICP-OES measurements were performed in instrumental triplicate. Phenol-removal experiments were performed in duplicate for each independent sample. Kinetic data for dye and phenol adsorption were fitted using pseudo-first-order and pseudo-second-order models, and the quality of fitting was assessed using the coefficient of determination (*R*^2^).

#### 2.2.6 Machine learning applications

A dataset was compiled comprising the physicochemical and functional characterisation data of magnetite-clay composite materials synthesised *via* different techniques. Each sample was associated with multiple numerical descriptors (*e.g.*, specific surface area, zeta potential, pH_nat_, d_DLS_, Fe_3_O_4_ real amount (%), pH_IEP_, *etc.*). The target variable (adsorption as milligrams of material, either dye or heavy metal, adsorbed per gram of sample) represents the sample's adsorption capacity (functionality). Two categorical variables, *i.e.*, Material_adsorption (indicating the adsorbed type, *e.g.*, Cu^2+^, Fe^3+^, RhB, MB, MO), and synthesis (describing the synthesis technique used), were preserved using one-hot encoding *via pandas.get_dummies* to enable integration into regression modelling.

##### 2.2.6.1 Data processing and analysis

Data pre-processing was performed in Python 3.10 using *pandas*, *numpy*, and *scikit-learn*.^42^ Missing values were addressed using Multivariate Imputation by Chained Equations (MICE) *via* the *IterativeImputer from sklearn. experimental*.^43^ This method iteratively fits each incomplete feature as a function of other features, leveraging linear regression in a round-robin fashion. All numeric columns, including both physicochemical and functional data, were imputed to ensure model readiness without discarding valuable samples. The column material (sample name) was excluded from further analysis due to the associated numerical representation (physicochemical and magnetic functional characterisation). Before modelling, all continuous features were normalised using z-score standardisation (*StandardScaler*) to ensure uniform feature scaling across the input space.

##### 2.2.6.2 Neural network training

We implemented a fully-connected feedforward neural network (multilayer perceptron, MLP^1^) using TensorFlow 2.15 and Keras. The architecture consisted of:

(i) An input layer with dimensionality matched to the number of post-imputation features.

(ii) A first hidden layer with 32 neurons with ReLU activation.

(iii) A second hidden layer with 16 neurons with ReLU activation and,

(iv) An output layer with a single neuron and linear activation for continuous regression.

**Fig. 2:**
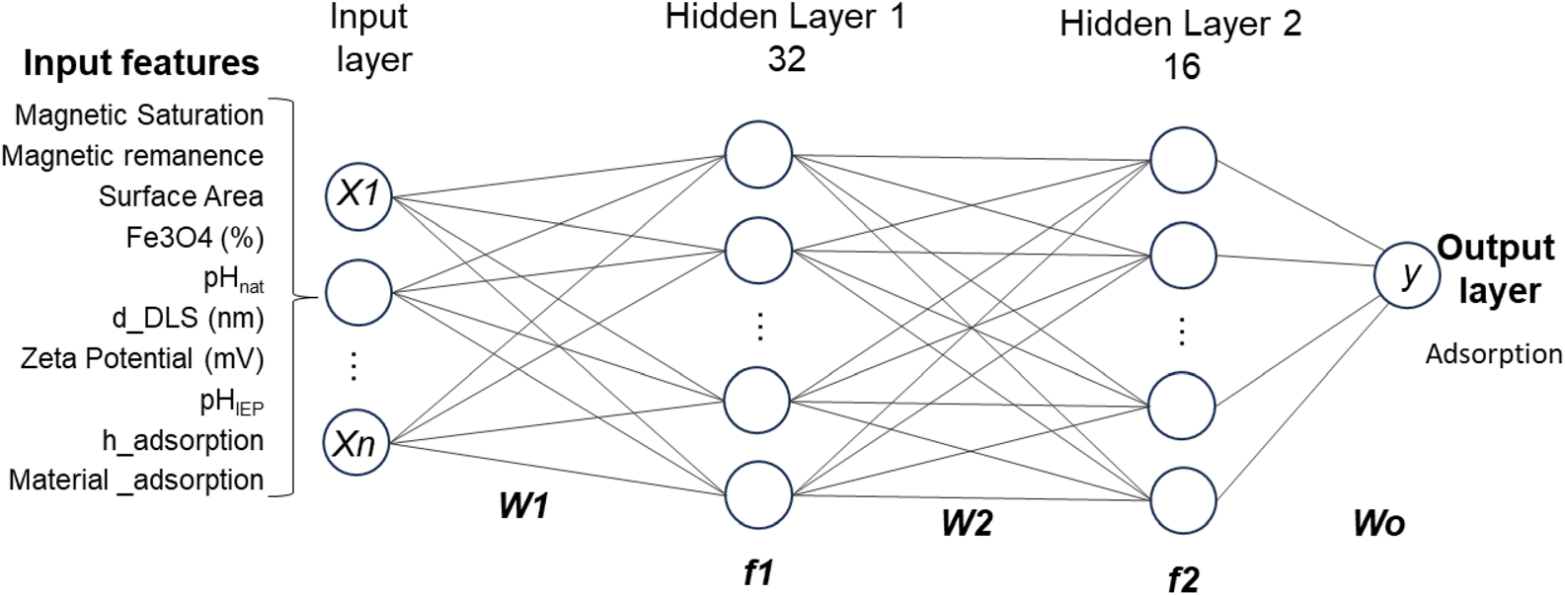
Schematic representation of the fully-connected feedforward neural network used for adsorption prediction, including an input layer of features (*x*_1_, *x*_2_, …, *x*_*n*_), followed by two hidden layers: the first with 32 neurons (activation function *f*_1_) and the second with 16 neurons (activation function *f*_2_), with corresponding weight matrices *W*_1_ and *W*_2_. The output layer comprises a single neuron predicting the adsorption capacity (*y*).

In this type of architecture, each neuron in one layer is connected to every neuron in the next, with learnable weights and biases defining the linear transformation: = *W*_*x*_ + *b*_*y*_ = where *W* is the weight matrix, *x* is the input vector, and *b* is the bias vector (Fig. 2).

The model was compiled using a loss function (Mean Squared Error (MSE)), and an Adam optimiser with a learning rate of 0.01. Model validation was performed using a hold-out test set and 5-fold cross-validation. First, the dataset was divided into training and test subsets using an 80/20 split with a fixed random seed (random state = 42). The model was trained on the training subset for 100 epochs with a batch size of 16. During training, 20% of the training data was used internally as a validation subset to monitor model convergence and overfitting. After training, the final model was evaluated on the held-out test subset using mean absolute error (MAE), MSE, and coefficient of determination (*R*^2^). To further assess model robustness, 5-fold cross-validation was also performed using shuffled folds with the same random seed. For each fold, a new neural network with the same architecture and hyperparameters was trained, using 20% of the corresponding training fold as an internal validation subset. The model was then evaluated on the corresponding test fold. The final cross-validation performance was reported as the mean ± standard deviation of MAE, MSE, and *R*^2^ across the five folds.

##### 2.2.6.3 Feature importance

To gain insights into the contributions of individual input variables toward the prediction of adsorption performance, we employed model-derived and model-agnostic feature importance techniques across three different regression models: a Random Forest Regressor, a Gradient Boosting Regressor, and a regularised linear Ridge Regression model. Feature importance scores were directly extracted from trained Random Forest and Gradient Boosting models, reflecting the relative contribution of each feature to reducing prediction error across all splits in the ensemble. These scores provide an interpretable, heuristic measure of how often and effectively each feature was used during the tree construction process. For the linear Ridge model, we applied permutation feature importance(Fisher *et al.*, 2019). This technique quantifies feature relevance by measuring the decrease in model performance (in terms of *R*^2^) when the values of a single feature are randomly permuted, thereby disrupting its relationship with the target variable. Bar and Bubble plots were generated to visualise and compare the importance rankings across models.

## 3 Results and discussion

### 3.1 Physical–chemical characterisation

To elucidate the structural and functional features of the clay/Fe_3_O_4_ composites obtained by heterocoagulation and coprecipitation followed by spray-drying, we performed a comprehensive physicochemical characterisation. Particular attention was given to colloidal stability, crystalline structure, morphology, surface area, and magnetic behaviour, as these parameters critically determine the performance and potential applications of the composites.

#### 3.1.1 Heterocoagulated samples (HC)

**Fig. 3:**
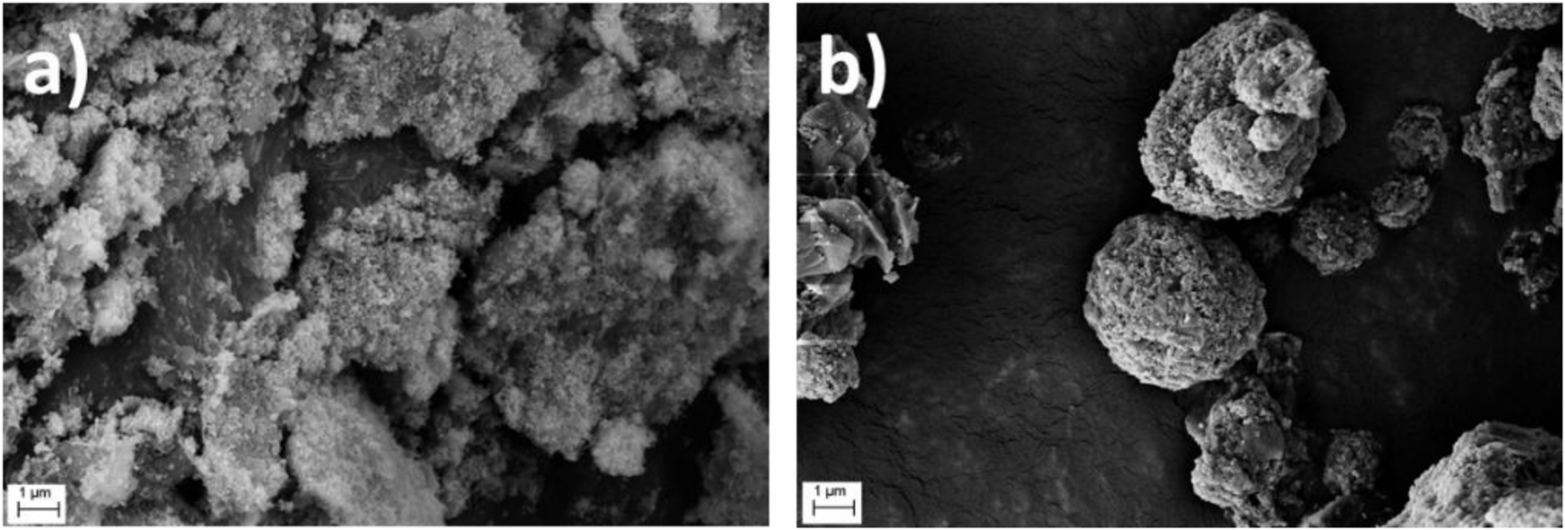
SEM photos of: (a) CaB/Fe_3_O_4__1 : 1_HC dried from suspension and (b) CaB/Fe_3_O_4__1 : 1_HC granules obtained *via* spray drying granulation process at 20.000× magnification.

Colloidal characterisation data of pure clays and heterocoagulated samples are reported in Table S2. Fig. S1 and S2 show the Dynamic Light Scattering (DLS) diameter size distribution (intensity-weighted and integrated by number) of NaB/Fe_3_O_4_ heterocoagulated samples, compared with single components. The general trend also observed for other HC samples is that composites did not show the typical, uniformly sized granulated structure.^44^ Rather, the data reflect the size distribution of the individual components, with the nanometric magnetite not significantly affecting the size of the coarser clay particles. The zeta potential *vs.* pH curves of the individual components and the HC composites (Fig. S3) show that the Fe_3_O_4_/clay composites follow a trend similar to that of the corresponding clays. The cationic clays, as well as the corresponding composites, displayed a negative zeta potential (ranging from −8 mV to −25 mV) that ensures a good colloidal stability, in line with results reported in literature. On the contrary, the anionic clay LDH2 and the corresponding composites exhibited a basic pH_IEP_, very close to their natural pH (8/9), which accounts for the limited stability of these samples. The comparison of XRD patterns of HC composites and single components (Fig. S4–S6), with a focus on the *d*_001_ crystal plane (peak at low angles, <10°), allowed us to investigate potential shifts resulting from interactions with magnetite. We found that only the MMT and NaB composites exhibited a slight shift of the *d*_001_ peak to lower angles, with a calculated increase in interlayer spacing, from about 1.25 nm to 1.45 nm. This result can be attributed to the high cation exchange capacity of montmorillonite and sodium bentonite,^45,46^ which facilitates the intercalation of magnetite into the clay layers, in some cases leading to partial delamination of the clay structure (disappearance of 001 peak for MMT 1 : 1 sample).^47,48^ In all other samples, we can only hypothesise that Fe_3_O_4_ physically adsorbed onto the clay surfaces, which did not appear to alter the crystalline structure or significantly affect the colloidal behaviour, as previously discussed. More specifically, the XRD pattern of pure Fe_3_O_4_, characterised by diffraction peaks at 30.1°, 35.4°, 43.1°, 53.4°, 56.9°, and 62.5°, was also observed in all clay-based composites, together with additional reflections corresponding to the pristine clays.^49^ MMT-based composites exhibited characteristic peaks at 7.1° (001), 19.8° (110), and 34.9° (110).^50,51^ LDH2 composites showed two intense and sharp reflections at 11.6° (003) and 23.5° (006), along with less intense peaks at 35.4° (012), 40.1° (015), 47.5° (018), 61.6° (110), and 62.9° (113).^52,53^ NaB and CaB composites displayed similar diffraction patterns, with peaks at 5.8° and 19.8° assigned to the montmorillonite phase, a peak at 26.6° corresponding to quartz, and a reflection at 21.9° related to the albite phase.^54^ In addition, NaB showed two extra peaks at 32° and 46°, attributed to the sodium chloride phase.^55^ Finally, sepiolite-based composites presented several characteristic reflections at 7.3° (110), 19.1° (060), 20.8° (131), 23.3° (080), 27.1° (202), and 35.3° (541), confirming the preservation of the sepiolite crystal structure.^56^ The morphologies of the spray-dried CaB/Fe_3_O_4__1 : 1_HC sample and, for comparison, of the corresponding oven-dried sample are shown in Fig. 3. Overall, the formation of a granulated structure by spray-drying is confirmed with the formation of micrometric particles (2–10 µm) with various morphologies depending on the type of clay used as precursor. Irregular, spherical, lamellar-like, and wire-shaped granules were observed. Compared to the hydrodynamic sizes of dispersed samples before spray-drying (Table S2), the particle dimensions increased by approximately five to ten times, making the granulated powder easier to handle. The presence of Fe_3_O_4_ nanoparticles, in general, was confirmed through imaging and EDS analysis. Predominantly, small bright Fe_3_O_4_ nanoparticles and aggregates were observed on the surface of the clay-based granules. EDS analysis performed on individual granules further confirmed the presence of iron, clearly distinguishing it from gold (Fig. S7). Finally, ICP quantitative analysis confirmed the presence of magnetite in an amount almost consistent with the theoretical content (Table S3).

**Fig. 4:**
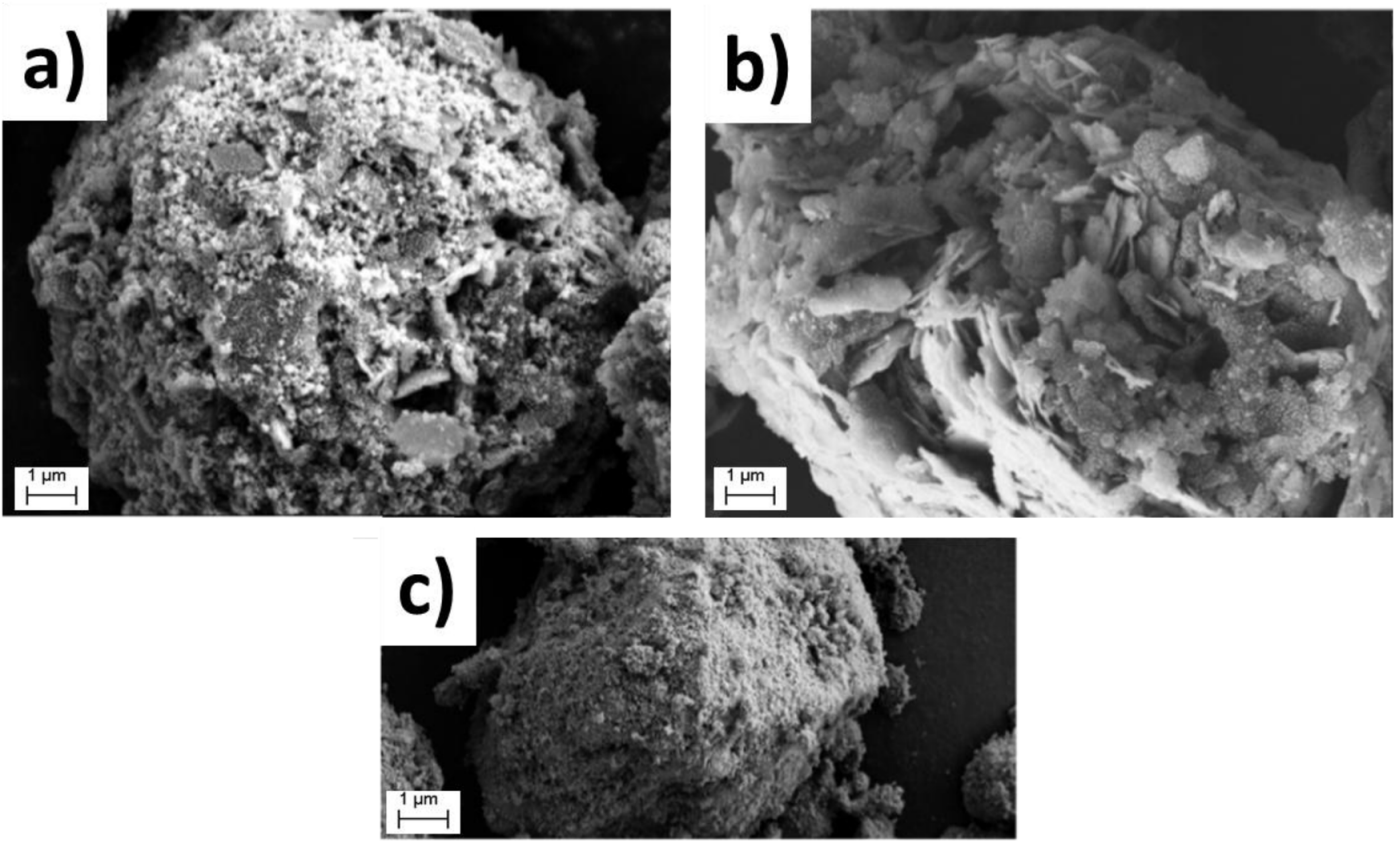
SEM images of Fe_3_O_4_ granules (a) and (b) embedded in LDH2 matrix at LDH2 : Fe_3_O_4_ 1 : 1 and LDH2 : Fe_3_O_4_ 5 : 1, respectively, and (c) in their pristine form.

The morphology of the LDH2/Fe_3_O_4__HC composites (Fig. 4a and b) is compared with that of pristine Fe_3_O_4_ (Fig. 4c). The SEM image of pristine Fe_3_O_4_ shows typical aggregated, nanostructured granules formed by nanoparticles strongly adhering to each other. In contrast, the SEM images of the 1 : 1 and 5 : 1 composites clearly display nanometric Fe_3_O_4_ particles distributed over the surface of plate-like clay aggregates, with the 5 : 1 sample exhibiting a markedly improved dispersion as a result of clay being the predominant phase.

Finally, comparing the data of specific surface reported in Table S4, we observed that HC composites showed a surface area comparable to or even higher than that of the corresponding components. Mainly, magnetic nanoparticles sticking onto the clays' surface and between their sheets may cause an increase in either the composites' area-to-volume ratio or in the clays' particles dispersion.^57,58^ In addition, SD granulation may cause an increase in SSA by producing more spherical particles (greater area-to-volume ratio) and by reducing the amount of clay agglomerates, as reported in the literature.^59^

#### 3.1.2 Coprecipitated samples (CP)

**Fig. 5:**
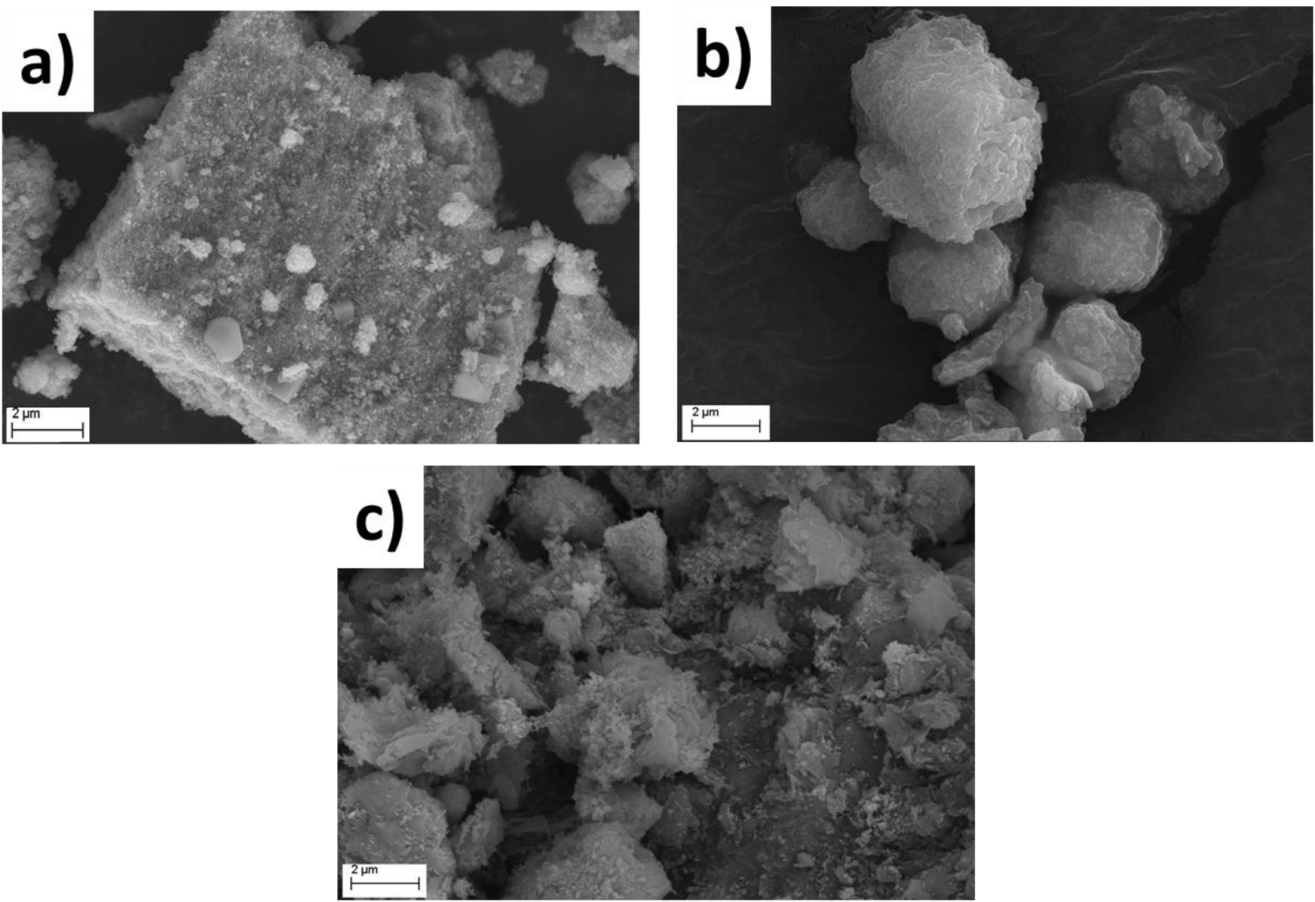
SEM photos of: (a) Fe_3_O_4__CP, (b) CaB@Fe_3_O_4__3 : 1_CP after and (c) before the spray drying granulation process at 20 000× magnification.

The colloidal characterisation of CP samples is summarised in Table S5. The hydrodynamic sizes of the co-aggregates were larger than those of the pristine clays, confirming the formation of bigger clusters due to aggregation and growth phenomena occurring during coprecipitation. Coprecipitated magnetite (Fe_3_O_4__CP) showed a pH_IEP_ of ∼7, comparable to commercial magnetite (Fig. S8) and consistent with literature values.^60,61^ Cationic clay-composites exhibited negative zeta potential values across the entire pH range (Fig. S9), indicating that the exposed clay surface is not fully covered by Fe_3_O_4_ nanoparticles, which carry a positive net surface charge below their pH_IEP_ (7.5). Similarly, the anionic LDH2 composite exhibited an IEP close to that of pure LDH2 (around pH = 9), confirming that the amount of magnetite present is insufficient to neutralise or invert the net surface charge of LDH2. The X-ray diffractograms (Fig. S10) of coprecipitated magnetite (Fe_3_O_4__CP) exhibit several peaks positioned between those of maghemite and magnetite at 30.2° (220), 35.7° (311), 43.1° (400), 53.7° (422), 57.4° (511) and 62.9° (400). However, the nanoparticle peaks of the Clay@Fe_3_O_4__CP composites shift to slightly lower 2*θ* values, suggesting the formation of the Fe_3_O_4_ crystal phase only, which diffracts at lower angles than γ-Fe_2_O_3_.^62^ Additionally, the main clay peaks previously described in section 3.1.1 undergo a slight shift to lower angles upon the formation of the magnetic coprecipitated composites, likely due to the intercalation of Fe_3_O_4_ nanoparticles.^63^ SEM-FEG analysis of coprecipitated samples before and after spray drying is shown in Fig. 5. As seen in Fig. 5a, Fe_3_O_4__CP formed large aggregates owing to the absence of dispersing or capping agents during synthesis, which limits control over particle growth. Oven-dried composites (Fig. 5c) also exhibited microsized aggregates (2–10 µm) with heterogeneous morphologies, including irregular, lamellar-like and wire-like structures. The spray-granulation process (Fig. 5b) successfully spheroidised these aggregates, yielding more compact and rounded granules.

Diffuse iron-rich regions detected on the micrometric granules confirm the presence of surface nanoparticles, as supported by EDS analysis (Fig. S11). SSA values for CP samples are provided in Table S6. Coprecipitated magnetite showed a significantly higher SSA (78 m^2^ g^−1^) compared to commercial magnetite (7 m^2^ g^−1^), consistent with literature trends.^64–66^ CP composites also exhibited higher SSA values than HC composites, except for MMTK10 and sepiolite-based systems, in which the inherently high SSA of the pristine clays likely decreased upon Fe_3_O_4_ precipitation due to partial surface and pore coverage, as previously reported.^67,68^

### 3.2 Functional characterisation

#### 3.2.1 Magnetic characterisation

**Fig. 6:**
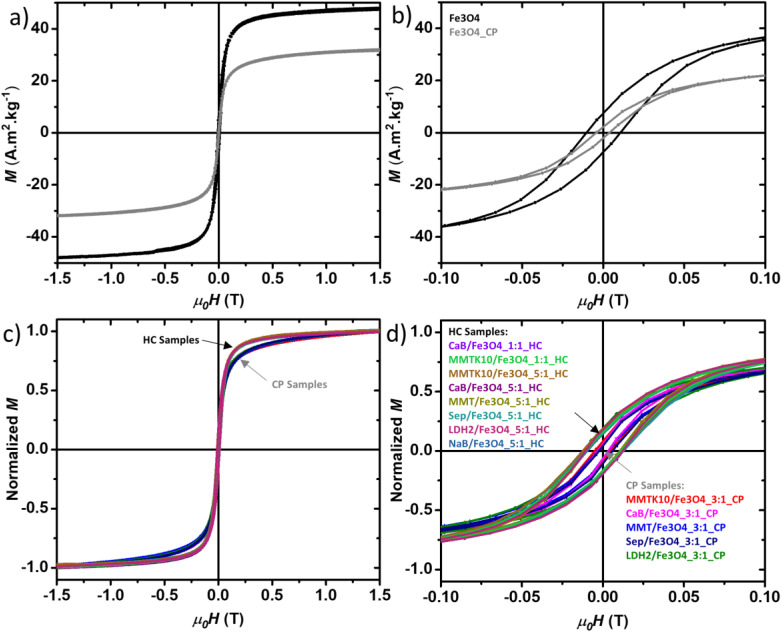
Magnetisation loops at room temperature for Fe_3_O_4_ and Fe_3_O_4__CP (a) in the full range *µ*_0_H = ±1.5 T, (b) a zoom in the loops showing the field range *µ*_0_H = ±100 mT. Normalised magnetic loops of the composites, (c) in the full range *µ*_0_H = ±1.5 T, and (d) a zoom in the loops showing the field range *µ*_0_H = ±100 mT.

We measured and analysed the magnetisation (*M*) *vs.* magnetic field (*µ*_0_H) curves of all samples at room temperature (Fig. 6). The commercial Fe_3_O_4_ showed a saturation magnetisation of *M*_s_ = 49.9 Am ^2^ Kg ^−1^ at *µ*_0_H = 1.5 T, with a magnetic remanence *M*_r_ = 0.009 Am ^2^ Kg ^−1^ and a magnetic coercive field of *µ*_0_*H*_c_ = 20 mT, whereas the coprecipitated Fe_3_O_4_ NPs showed *M*_s_ = 39.5 Am ^2^ Kg ^−1^ at *µ*_0_H = 1.5 T, *M*_r_ = 0.006 Am ^2^ Kg ^−1^ and *µ*_0_*H*_c_ = 3 mT (Fig. 6a and b). The composite samples (Fig. 6c and d) also showed loops similar to those of the parent Fe_3_O_4_ NPs samples, confirming that the chemical and thermal treatments during the composite production did not significantly modify the magnetic properties of the Fe_3_O_4_ NPs in the samples. The HC samples also displayed a sharper increase in magnetisation with field compared to CP samples, resulting in a visible gap around *µ*_0_H = 250 mT, which is again the footprint of the Fe_3_O_4_ NPs from which they have been composed.^69^

#### 3.2.2 Heavy metal adsorption test

The adsorption capacity of pure clays and heterocoagulated composites (Table S7) followed comparable trends, mainly dictated by the clay : Fe_3_O_4_ ratio. Higher clay fractions consistently yielded greater removal efficiencies, reaching adsorption capacities up to 8.6 mg g^−1^ for MMT/Fe_3_O_4__5 : 1_HC, driven by increased opportunities for electrostatic interactions, cation-surface complexation, and ion-exchange within the clay layers.^70,71^ Among the tested pure clays, the superior performance of MMT and CaB in Cu^2+^ (up to 9.3 mg g^−1^) and Fe^3+^ (up to 15.9 mg g^−1^) removal can be attributed to their smectite layered structure, which provides a high cation exchange capacity and accessible, expandable interlayers.^72^ These features enable efficient uptake of multivalent metal ions predominantly through ion-exchange and inner-sphere complexation mechanisms. In particular, NaB, despite its higher swelling capacity, may suffer from excessive layer expansion that reduces effective site confinement and weakens metal binding under dynamic conditions. Non-smectitic or structurally constrained materials, such as Sep, primarily rely on surface silanol interactions and thus exhibit lower adsorption capacities due to the absence of true interlayer exchange sites. Similarly, LDH2 is intrinsically more selective toward anionic species, resulting in limited affinity for cationic pollutants such as Cu^2+^ and Fe^3+^. Finally, surface-modified MMT systems (MMTK10) may experience partial blockage of exchange sites, reducing the accessibility of active sites and consequently decreasing overall metal uptake.^73,74^ These conclusions can also be made for the respective clay composites. In fact, Fe_3_O_4_ did not impart any synergistic improvement, although a minor increase in Cu^2+^ uptake was occasionally observed. Coprecipitated composites showed much lower adsorption capacities than the heterocoagulated ones (Table S8). Fe^3+^ removal decreased only slightly, while Cu^2+^ adsorption dropped sharply. This reduced performance is likely due to the closer interaction between clays and coprecipitated magnetite, which can block surface groups and reduce the clay's cation-exchange ability, limiting electrostatic attraction with Cu^2+^. In both HC and CP samples, the adsorption equilibrium was reached within the first hour and remained stable up to 24 h. Although kinetic studies were not performed during the metal adsorption experiments, literature reports indicate that metal cations are predominantly removed through chemisorption processes, as supported by the good agreement of literature data with the pseudo-second-order kinetic model. This suggests that the removal mechanism is governed by specific interactions between the metal ions and the active sites of the clay, including ion exchange with interlayer cations and complexation with surface hydroxyl groups. In particular, metal ions can replace exchangeable cations within the clay structure while also forming inner-sphere complexes with hydroxyl groups located at the edges and surfaces of the clay minerals.^75,76^ From the reusability tests performed for Cu^2+^ removal (Fig. S12), CaB/Fe_3_O_4__5 : 1_HC exhibited a drastic decrease in removal efficiency over four consecutive cycles, losing approximately 50% of its initial adsorption capacity. This reduction may be attributed to the progressive saturation of active sites, as the adsorbed Cu^2+^ ions were not desorbed between cycles. In contrast, LDH2/Fe_3_O_4__5 : 1_HC showed excellent stability, with only a 5% reduction in performance.

#### 3.2.3 Dye adsorption evaluation and kinetic studies

The dye removal efficiencies of pristine clays, heterocoagulated, and coprecipitated co-aggregates are summarised in Tables S9 and S10. Negatively charged clays (all except LDH2) and their HC analogues (*ζ* < 0 mV at pH_nat_ and pH_IEP_ < 2) efficiently removed the cationic dyes RhB and MB, with MB showing the highest uptake (typically >90%), reaching adsorption capacities up to 19.7 and 7 mg g^−1^ for RhB and MB, respectively. Both HC and CP composites outperformed the individual components, attributed to FeO^−^ groups that strengthen electrostatic attraction between cationic dyes and the permanent negative charges of clays.^65,70^ This synergistic effect has been reported previously for Fe_3_O_4_-clay systems.^57,77^ Conversely, LDH2-based composites, bearing a positive surface charge (*ζ* > 0 mV at pH_nat_ and pH_IEP_ > 8) and exhibiting anion-exchange capacity, were highly effective toward MO, as expected, reaching adsorption capacities up to 6.6 mg g^−1.^^78,79^ Moreover, these materials exhibited lower adsorption capacities, likely due to their reduced surface area and ion-exchange capacity, which limit the availability of active adsorption sites. As observed for heavy-metal adsorption, anionic dye uptake was consistently higher in HC composites than in CP materials, with the latter often performing worse than the pristine clays. Kinetic analyses revealed that both pseudo-first-order and pseudo-second-order models fit the experimental data, although the latter predominated across all systems (Tables S11–S13). This indicates that, despite surface chemisorption governing the rate-limiting step, even physisorption affects the dye removal capacity, consistent with previously reported clay-dye adsorption mechanisms.^57,80^ Thus, in addition to electrostatic attraction toward oppositely charged dyes (*i.e.*, cationic MB and RhB for the negatively charged clays, and anionic MO for the positively charged LDH), clays and their respective co-aggregates exhibit a highly porous layered structure, high ion-exchange capacity, and pronounced swelling behaviour. These features enable not only ion-exchange-driven uptake but also sterically assisted adsorption, as the expanded interlayer spacing can accommodate nanoscale dye molecules, enhancing their overall retention within the clay structure. Notably, while both are cationic, the slightly lower uptake of RhB compared to MB can be attributed to its bulkier molecular structure, which experiences greater steric hindrance when accessing the narrow interlayer sites compared to the more planar MB molecule. Overall, clay-Fe_3_O_4_ composites showed excellent dye removal performance under the tested conditions when compared with other clay-based or Fe_3_O_4_-based composite materials reported in the literature.^81^ Nevertheless, the removal capacity was found to be strongly dependent on the specific adsorbent material used as support for magnetite.^82^ From the reusability tests (Fig. S13a–b), the CaB/Fe_3_O_4__5 : 1_HC co-aggregate showed a gradual decrease in removal efficiency for both MB (from 27.4 to 25.0 mg g^−1^) and RhB (from 26.2 to 23.8 mg g^−1^) over successive cycles. A similar trend was observed for the LDH2/Fe_3_O_4__5 : 1_HC system, which exhibited a reduction in MO removal efficiency from 29.8 to 27.6 mg g^−1^. This progressive decline in performance can be mainly attributed to the partial saturation and/or blockage of active sites on the surface of the co-aggregates. During repeated adsorption cycles, the accumulation of residual dye molecules likely hinders the accessibility of active sites, thereby reducing the overall adsorption capacity.

#### 3.2.4 Phenols removal evaluation and kinetic studies

The best-performing materials for heavy-metal and dye removal were also evaluated for phenol adsorption. Both CaB/Fe_3_O_4__5 : 1_HC and LDH2/Fe_3_O_4__5 : 1_HC composites showed significant activity. LDH2/Fe_3_O_4__5 : 1_HC displayed the highest performance, reaching equilibrium after 3 h with a phenol uptake of 43.99 µg g^−1^, substantially higher than the individual components, LDH2 (8.8 µg g^−1^) and Fe_3_O_4_ (23.3 µg g^−1^) after 24 h. CaB/Fe_3_O_4__5 : 1_HC reached equilibrium after 48 h, achieving an adsorption capacity of 30.83 µg g^−1^, again markedly outperforming bare bentonite, which removed only 0.8 µg g^−1^ after 24 h. Kinetic parameters are summarised in Table S14. Phenol adsorption followed the pseudo-second-order model predominantly, with high correlation coefficients (*R*^2^ = 0.988 for CaB-based and 0.993 for LDH2-based composites). The dominance of chemisorption as the rate-limiting step is consistent with the absence of electrostatic interactions in phenol uptake (pseudo-first order kinetic model *R*^2^ < 0.5), which is governed instead by electron-exchange and sharing mechanisms, in agreement with previous reports.^83–85^ From a structural perspective, LDH2 and its composite exhibit enhanced phenol removal efficiency, likely due to the combined contribution of intercalation and surface adsorption mechanisms. In contrast, conventional bentonite shows comparatively lower adsorption capacity, reflecting its limited affinity toward neutral organic molecules.

#### 3.2.5 Machine learning applications

A dataset of 350 rows and 13 features was compiled, comprising the physicochemical and functional characterisation data of magnetite-clay composite materials synthesised *via* different techniques and the adsorption functional measurements (Table 1). The dataset was structured according to the material adsorption feature, wherein each composite material was tested against multiple analytes (*e.g.*, Cu^2+^, Fe^3+^, RhB, MO, MB). For each analyte, functional adsorption data were recorded at specific time points (1 h and 24 h), resulting in repeated rows for the same material with varying adsorption targets and durations. For instance, the first 144 entries correspond to the adsorption performance of each sample for Cu^2+^ at 1 h or 24 h. This design allowed the inclusion of time-dependent adsorption behaviour while preserving a consistent link between sample physicochemical and magnetic features.

**Table 1:** Dataset overview. The table summarises each feature included in the dataset, including a short description, data type, number of missing data points, and the corresponding minimum and maximum values

Features	Explanation	Data type	Missing values (count)	Min values	Max values
Material	Sample's names	Categorical	0.0		
Synthesis	Synthesis methods	Categorical	0.0		
Magnetic saturation	(Am^2^ Kg_Fe_3_O_4__^−1^)	Numerical	30.0	0.0	82.4
Magnetic remanence	(Am^2^ Kg_Fe_3_O_4__^−1^)	Numerical	30.0	0.0	8.6
Surface area	Specific surface area *via* Brunauer–Emmett–Teller (BET)	Numerical	0.0	7.0	250.0
Fe_3_O_4_ (%)	Real Fe_3_O_4_ percentage	Numerical	0.0	0.0	95.0
pH_nat_		Numerical	0.0	4.4	8.7
d_DLS (nm)	Hydrodynamic size *via* DLS	Numerical	0.0	55.0	1350.0
Zeta potential (mV)		Numerical	0.0	−34.0	31.0
pH_IEP_		Numerical	0.0	2.0	9.3
h_adsorption	Duration of adsorption test performed: 1 hour or 24 hours	Numerical	0.0	1.0	24.0
Material_adsorption	(*e.g.*, Cu^2+^, Fe^3+^, RhB, MB, MO)	Categorical	0.0		
Adsorption	Milligrams of material adsorbed per gram of sample	Numerical	0.0	0.1	99.9

#### 3.2.6 Neural network training

**Fig. 7:**
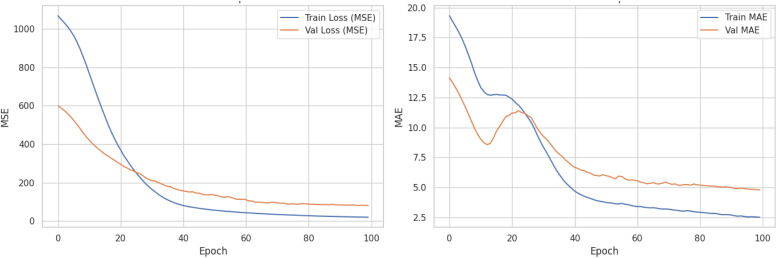
Training history of the neural network model for the hold-out training procedure. T. The left panel shows the MSE loss for the training and internal validation subsets over 100 epochs, both decreasing and plateauing after approximately epoch 60, indicating stable learning. The right panel displays the MAE evolution, where the training MAE drops toward ∼2.5, while the validation MAE converges around ∼6, suggesting good generalisation without overfitting.

To capture nonlinear relationships between the physicochemical properties of the samples and their adsorption capacity, an artificial neural network was trained using scaled data. Training was performed over 100 epochs using the Adam optimiser and mean squared error (MSE) as the loss function. The dataset was first divided into training and held-out test subsets using an 80/20 split. During training, 20% of the training subset was used as an internal validation set to monitor convergence over 100 epochs. Fig. 7 shows the training and validation histories, where both MSE and MAE decreased and reached a plateau, supporting stable learning without evident overfitting.

In addition to this hold-out evaluation, 5-fold cross-validation was performed to assess the robustness of the model across different data partitions. In each fold, the neural network was reinitialised and trained from scratch using the same architecture and hyperparameters. Across the five folds, the model achieved an average MAE of 4.32 ± 0.36, an average MSE of 58.4 ± 5.96, and an average *R*^2^ of 0.91 ± 0.03, indicating stable predictive performance and explaining more than 90% of the variance in adsorption capacity.

#### 3.2.7 Feature importance

**Fig. 8:**
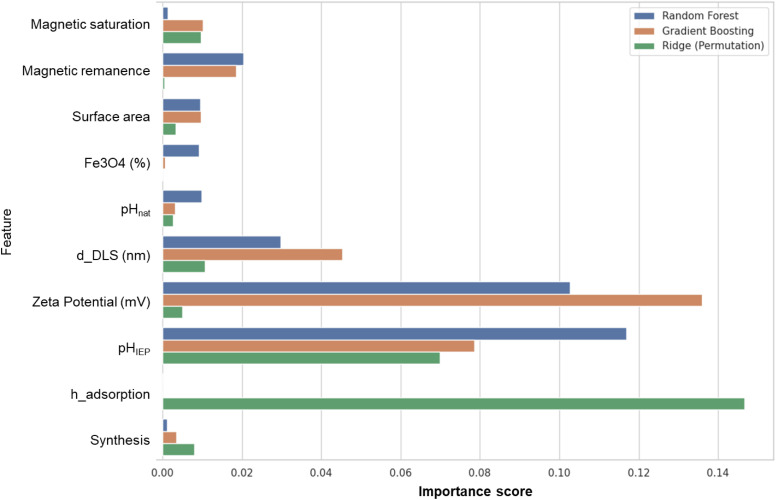
Comparative feature importance scores derived from three models: random forest (*R*^2^ = 0.95, blue), gradient boosting (*R*^2^ = 0.96, orange), and ridge regression (*R*^2^ = 0.78, green).

To investigate the contribution of individual physicochemical and functional features toward the prediction of adsorption capacity, we applied three complementary model-agnostic feature importance techniques: impurity-based importance from Random Forest and Gradient Boosting regressors, and permutation importance using Ridge Regression. As shown in Fig. 8, several features consistently emerged as important across the three models. Zeta potential and d_DLS (hydrodynamic size) exhibited high importance scores in both tree-based models. Additionally, pH_IEP_ (isoelectric point) and h_absorption (time-dependent adsorption behaviour) were highlighted across methods, suggesting the significance of surface charge transitions and adsorption kinetics in pollutant removal efficiency. Magnetic remanence and magnetic saturation contributed modestly in the tree-based models. Taken together, these results point toward a synergistic mechanism in which adsorption performance is governed by a combination of electrokinetic properties, particle size, adsorption kinetics, and magnetic responsiveness. The high relevance of zeta potential and pHIEP reflects the central role of surface charge in determining electrostatic affinity between the adsorbent and the target species. This agrees with the higher uptake of cationic dyes, such as MB and RhB, by negatively charged clay-based systems, and with the preferential adsorption of the anionic dye MO by the positively charged LDH2-based composites. Similarly, the importance of hydrodynamic size can be interpreted as an indirect descriptor of colloidal aggregation, particle accessibility, and exposed active surface, which may influence the availability of exchange and adsorption sites. The contribution of adsorption time is also consistent with the kinetic experiments, where several systems showed progressive uptake before reaching equilibrium. In contrast, surface area alone did not emerge as the dominant factor, supporting the experimental observation that high specific surface area does not necessarily translate into higher adsorption capacity when surface chemistry and pollutant charge compatibility are unfavourable. Therefore, the ML analysis does not replace the mechanistic interpretation derived from the adsorption experiments, but supports it by identifying the physicochemical descriptors most closely associated with the measured adsorption performance.

**Fig. 9:**
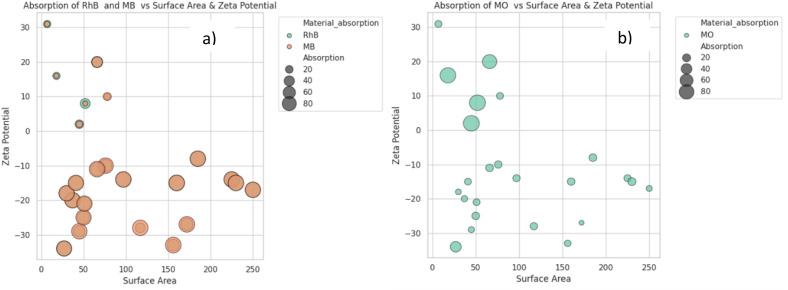
Bubble plots showing the relationship between surface area (*x*-axis), zeta potential (*y*-axis), and adsorption capacity (bubble size) for dye pollutants (a) RhB and MB; (b) MO.

To better capture the relationship between surface area, zeta potential and adsorption capacity (bubble size) for dye pollutants, bubble plots were used. The graphs reveal how surface area and surface charge interact in shaping dye adsorption, with distinct patterns emerging for different dye types (Fig. 9).

For cationic MB, strong adsorption was observed across a wide surface-area range, with all samples clustering in the zeta potential range (−10 to −30 mV), supporting the hypothesis that surface charge contributes significantly to adsorption capacity. Similarly, for anionic MO samples, higher adsorption is observed in those with a more positive zeta potential. It is noteworthy that very high surface areas (>200 m^2^ g^−1^) did not consistently correlate with high adsorption, underscoring the dominant role of surface chemistry in adsorption phenomena.

## 4 Conclusions

This work demonstrates a scalable route for the fabrication of magnetic clay-based composites with enhanced adsorption performance and practical suitability for water-remediation applications. The integration of Fe_3_O_4_ into cationic and anionic clays, achieved through heterocoagulation and coprecipitation, followed by spray drying, produced micrometric, free-flowing granules that preserved the structural integrity and surface functionality of the parent phases. The resulting materials exhibited strong removal efficiencies toward heavy metals, dyes, and phenolic pollutants, consistently outperforming the individual clays and enabling rapid magnetic recovery and reuse. Adsorption was governed by a combination of ion-exchange and electrostatic interactions, with contributions from both physisorption and chemisorption mechanisms. Machine-learning models trained on physicochemical and magnetic descriptors achieved high predictive accuracy (*R*^2^ ∼ 0.91), and explainability analyses identified zeta potential and pH_IEP_ as key drivers of performance. These insights provide mechanistic understanding and data-driven guidelines for the development of next-generation magnetic adsorbents tailored to specific classes of contaminants.

## Author contributions

Conceptualisation: Anna L. Costa, Maurizio Vespignani. Methodology: Simona Ortelli, Magda Blosi, Milad Takhsha. Investigation: Maurizio Vespignani, Ilaria Zanoni, Marina Naldi, Wendy Mensah, Alice Piraccini. Materials preparation and characterization: Maurizio Vespignani, Franca Albertini. Machine learning analysis: Irini Furxhi, Anna L. Costa. Data curation: Anna L. Costa, Ilaria Zanoni. Writing original draft: Maurizio Vespignani, Anna L. Costa. Writing, review and editing: all authors. Supervision: Anna L. Costa, Simona Ortelli, Magda Blosi.

## Conflicts of interest

There are no conflicts to declare.

## Supplementary Material

RA-016-D6RA01195K-s001

## Data Availability

The data supporting this article have been included as part of the supplementary information (SI). Supplementary information is available. See DOI: https://doi.org/10.1039/d6ra01195k.
