# Supplementary material for: Data-driven insights into the performance of scalable magnetic clay-based composites for pollutant removal
Source: RSC Adv. 2026 May 19;16(29):26833–46. doi: 10.1039/d6ra01195k (PMC13187916; doi:10.1039/d6ra01195k)
Supplement: RA-016-D6RA01195K-s001 [file RA-016-D6RA01195K-s001.pdf]

Supplementary Information (SI) for RSC Advances.
This journal is © The Royal Society of Chemistry 2026

Data-driven insights into the performance of scalable magnetic clay-based composites for pollutant removal

Maurizio Vespignani[1,2], Simona Ortelli[1*], Magda Blosi[1*], Ilaria Zanoni[1], Milad Takhsha[3], Franca Albertini[3], Irini Furxhi[1], Marina Naldi[4], Wendy Appiagyei Mensah[4], Alice Piraccini[4], Anna Luisa Costa[1].

[1] CNR-ISSMC, National Research Council of Italy-Institute of Science, Technology and Sustainability for Ceramics, Via Granarolo 64, 48018 Faenza, Italy.

[2] Department of Chemical Science, Life and Environmental Sustainability, Parma University, Parco Area delle Scienze, 11A, 43124 Parma, Italy.

[3] CNR-IMEM, National Research Council of Italy-Institute of Materials for Electronics and Magnetism, Parco Area delle Scienze, 37/A, 43124 Parma, Italy.

[4] Department of Pharmacy and Biotechnology, Alma Mater Studiorum University of Bologna, Via Belmeloro, 6, 40126 Bologna, Italy.

*Corresponding: simona.ortelli@issmc.cnr.it (S.Ortelli), magda.blosi@issmc.cnr.it (M. Blosi)

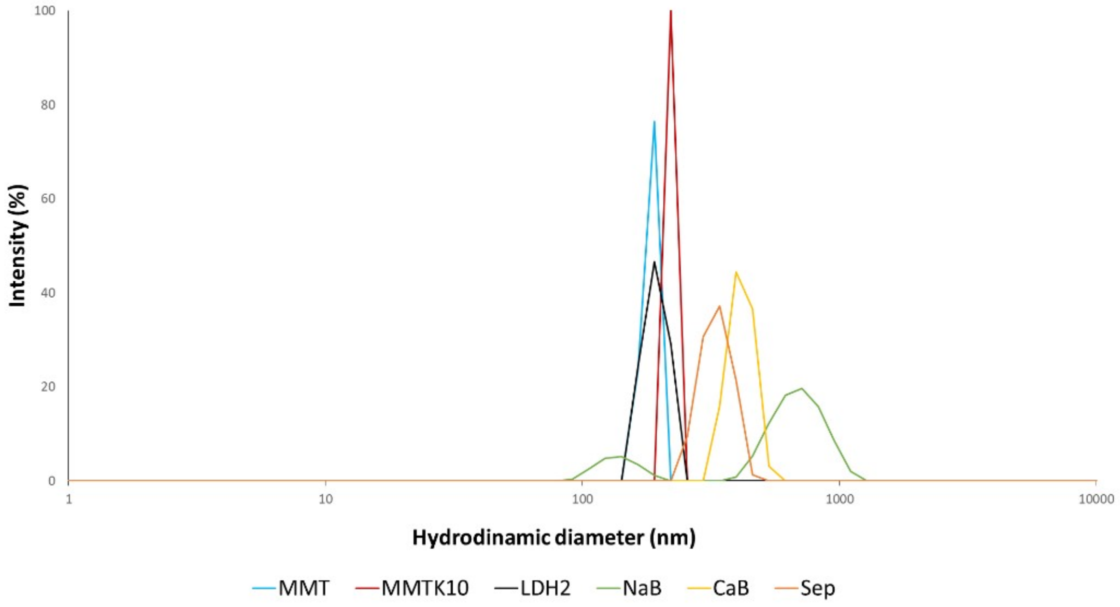

Figure S1 Hydrodynamic size distribution of pure clays in aqueous media at a concentration of 0.1g L^{-1} and a temperature of 25°C.

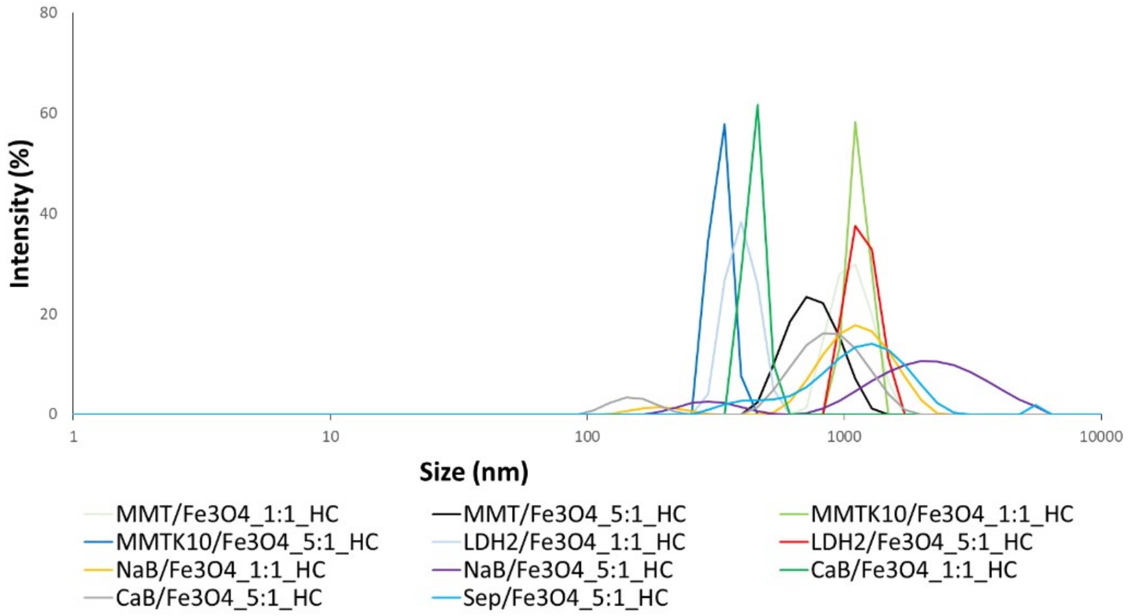

Figure S2 Hydrodynamic size distribution of different heterocoagulated samples in aqueous media at a concentration of 0.1g L^{-1} and a temperature of 25°C.

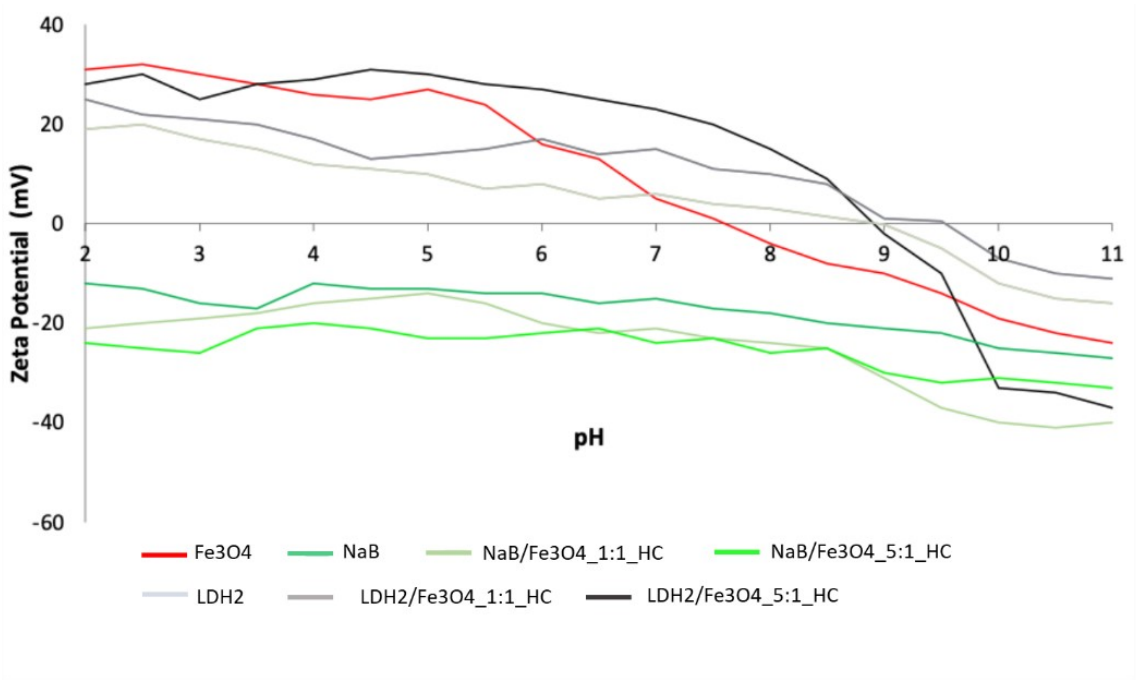

Figure S3 Zeta potential variation along the pH range for negatively-charged (NaB) and positively-charged (LDH2) heterocoagulated samples in aqueous media at a concentration of 0.1g L^{-1} and a temperature of 25°C.

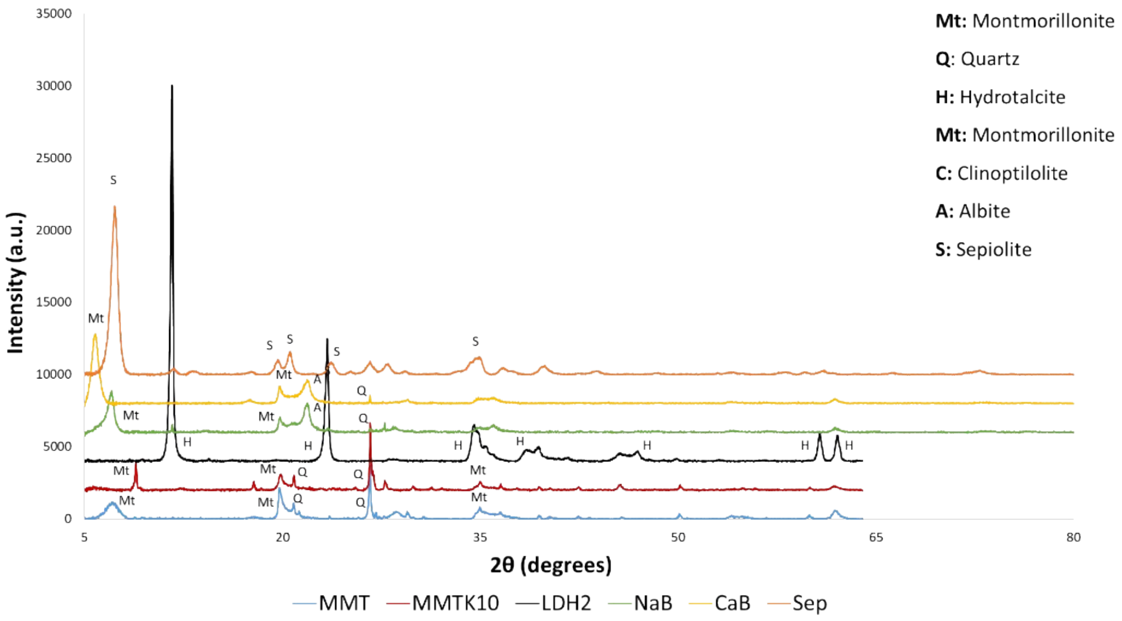

Figure S4 XRD diffractograms of pure clays recorded in the range 5-80 °2θ, counting for 0.5 sec every 0.02 °2θ step.

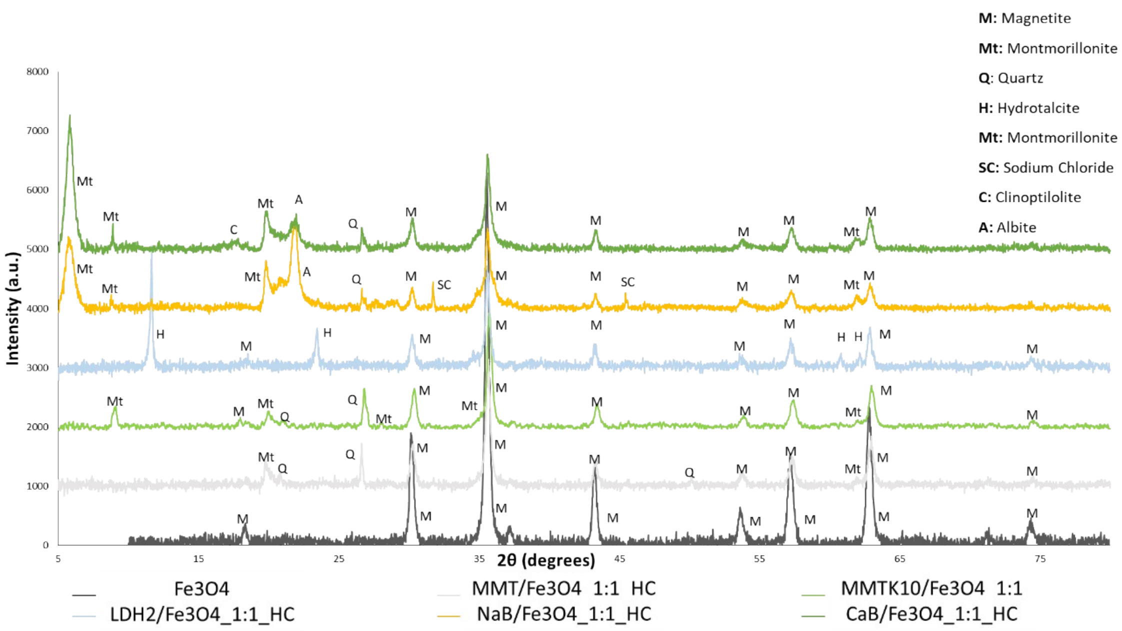

Figure S5 XRD diffractograms of magnetite and HC 1:1 samples recorded in the range 5-80 °2θ, counting for 0.5 sec every 0.02 °2θ step.

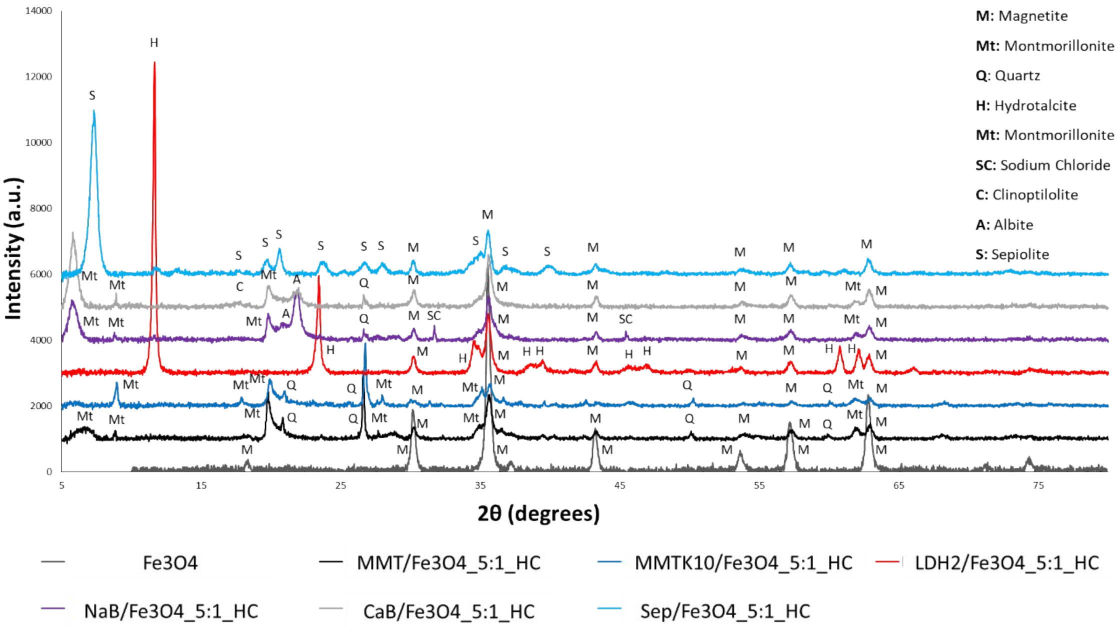

Figure S6 XRD diffractograms of magnetite and HC 5:1 samples recorded in the range 5-80 °2θ, counting for 0.5 sec every 0.02 °2θ step.

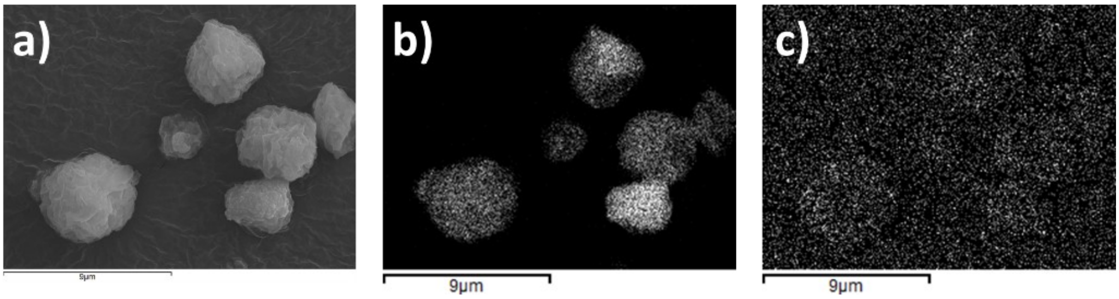

Figure S7 a) SEM photo of CaB/Fe_3O_4_1:1_HC; b) iron distribution within CaB/Fe_3O_4_1:1_HC identified through EDS map; c) gold distribution within CaB/Fe_3O_4_1:1_HC identified through EDS map.

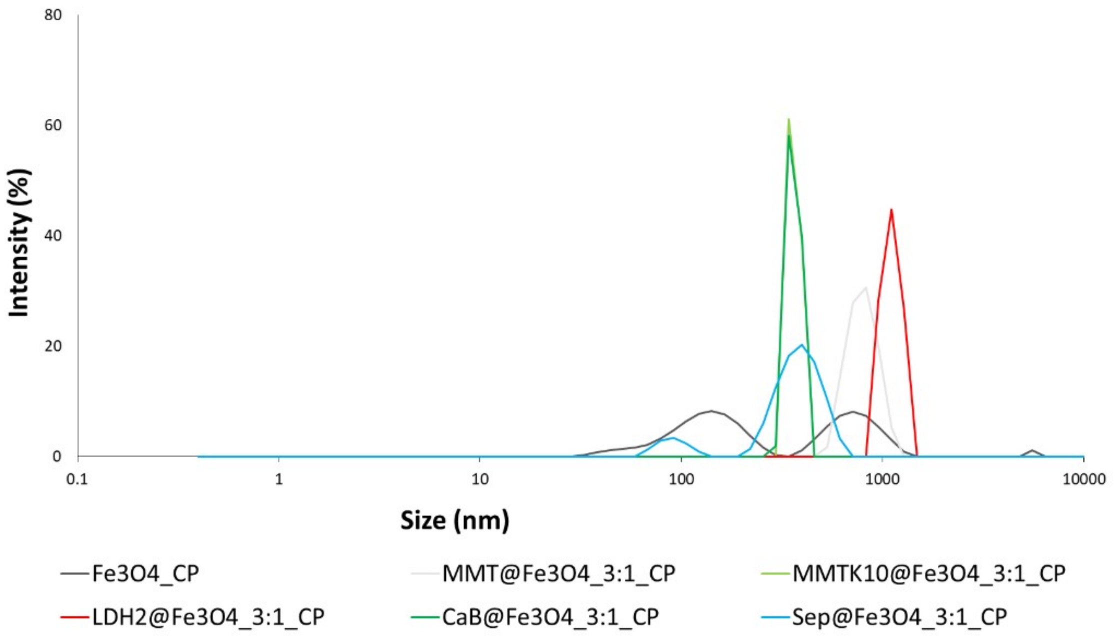

Figure S8 Hydrodynamic diameter distribution of different coprecipitated samples in aqueous media at a concentration of 0.1g L^{-1} and a temperature of 25°C.

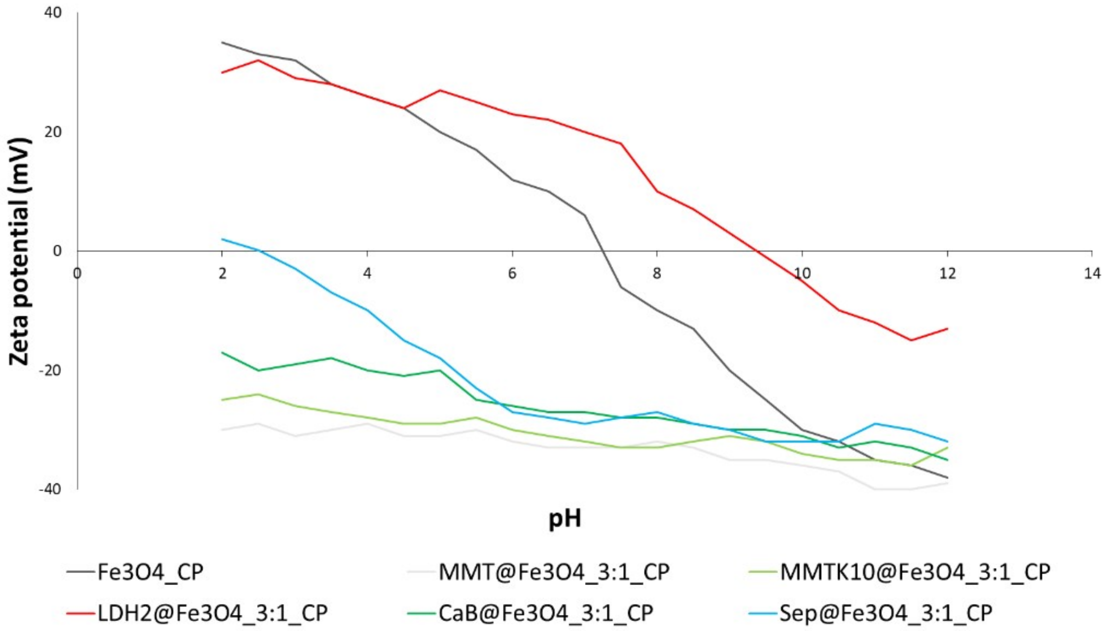

Figure S9 Zeta potential variation along the pH range for coprecipitated samples in aqueous media at a concentration of 0.1g L^{-1} and a temperature of 25°C.

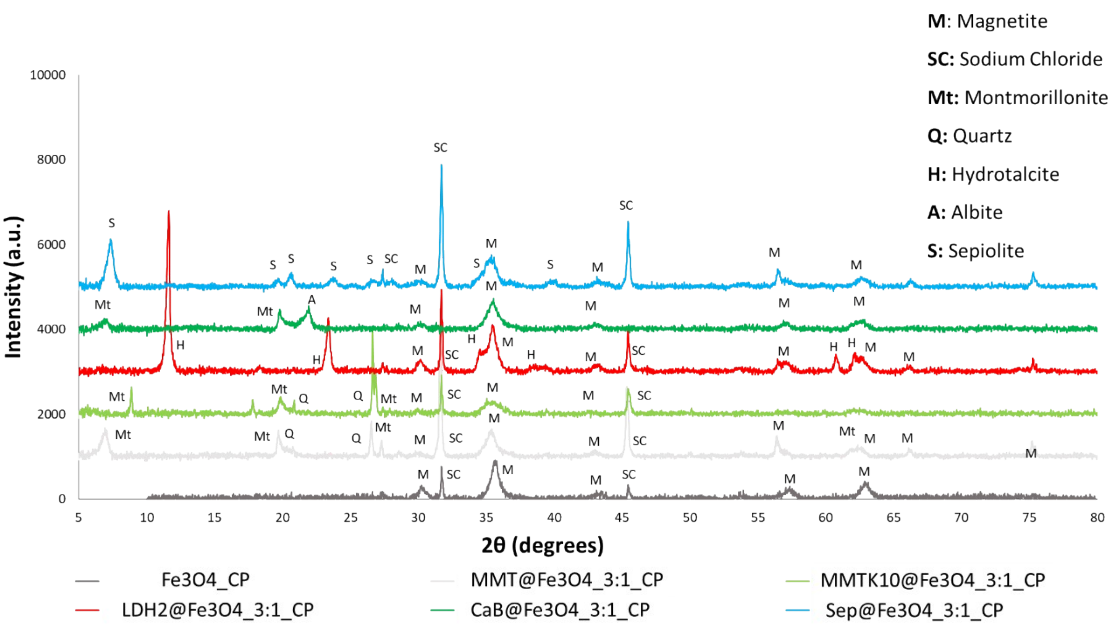

Figure S10 XRD diffractograms of coprecipitated samples recorded in the range 5-80 °2θ, counting for 0.5 sec every 0.02 °2θ step.

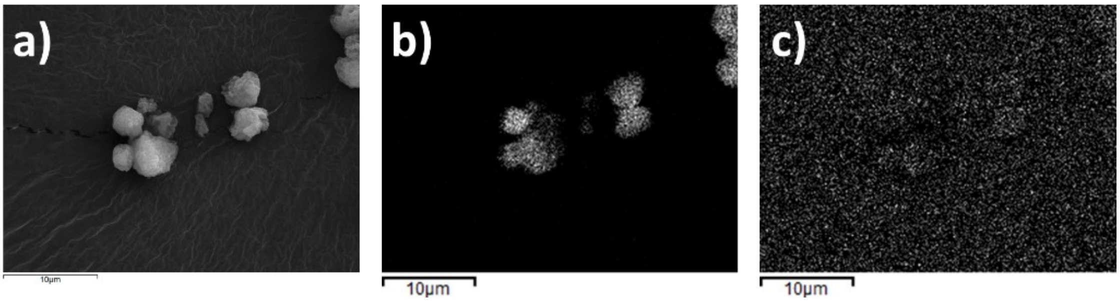

Figure S11 a) SEM photo of CaB@Fe_3O_4_3:1_CP; b) iron distribution within CaB@Fe_3O_4_3:1_CP identified through EDS map; c) gold distribution within CaB@Fe_3O_4_3:1_CP identified through EDS map.

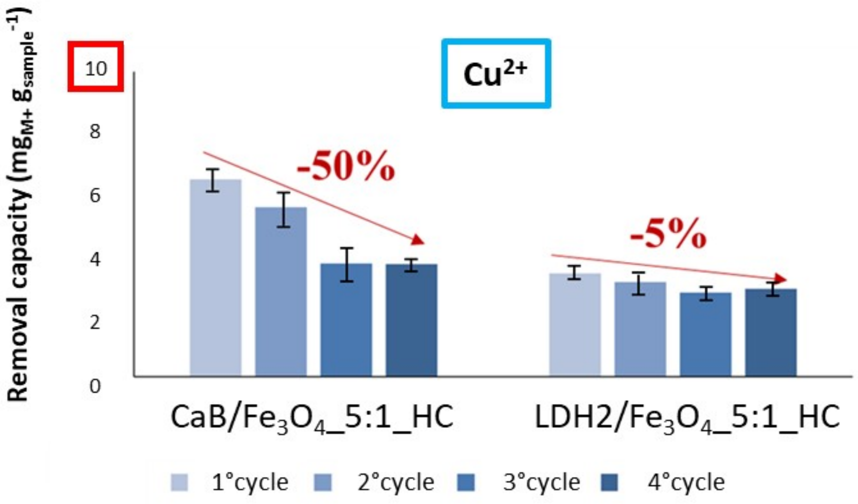

Figure S12 Cu^{2+} removal test along with 4 re-use cycles performed by using both CaB/Fe_3O_4_5:1_HC and LDH2/Fe_3O_4_5:1_HC samples.

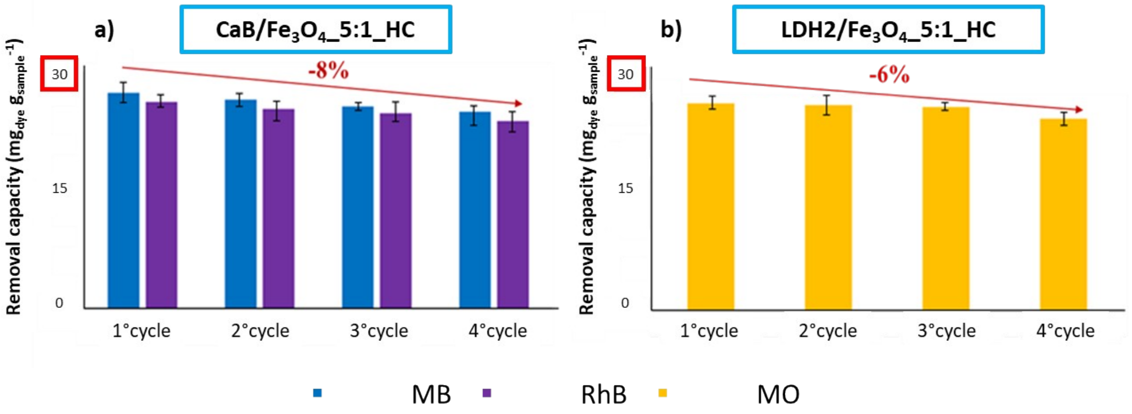

Figure S13 Dye removal test along with 4 re-use cycles performed by using a) CaB/Fe_3O_4_5:1_HC and b) LDH2/Fe_3O_4_5:1_HC samples.

Table S1 Fe_3O_4-clay composites synthesised via heterocoagulation (HC) and coprecipitation (CP).

Sample name	Heterocoagulation/Coprecipitation pH
MMT/Fe_3O_4_1:1_HC	5-6
MMT/Fe_3O_4_5:1_HC	5-6
MMTK10/Fe_3O_4_1:1_HC	5-6
MMTK10/Fe_3O_4_5:1_HC	5-6
LDH2/Fe_3O_4_1:1_HC	8-9
LDH2/Fe_3O_4_5:1_HC	8-9
NaB/Fe_3O_4_1:1_HC	5-6
NaB/Fe_3O_4_5:1_HC	5-6
CaB/Fe_3O_4_1:1_HC	5-6
CaB/Fe_3O_4_5:1_HC	5-6
Sep/Fe_3O_4_5:1_HC	5-6
Fe_3O_4_CP	10
MMT@Fe_3O_4_3:1_CP	10
MMTK10@Fe_3O_4_3:1_CP	10
LDH2@Fe_3O_4_3:1_CP	10
CaB@Fe_3O_4_3:1_CP	10
Sep@Fe_3O_4_3:1_CP	10

Table S2 Colloidal characterisation of heterocoagulated samples.

Sample name	pH_{nat}	d_{DLS} [a]	ζ [b]	pH_{IEP}
Fe_3O_4	4.4	55±5	31±2	7.5
MMT	6.8	515±95	-34±2	<2
MMTK10	4.8	320±40	-17±1	<2
LDH2	7.6	1160±160	16±2	8.9
NaB	6.7	125±25	-15±1	<2
CaB	7.2	410±60	-10±2	<2
Sep	8.3	320±55	-14±3	2.8
MMT/Fe_3O_4_1:1_HC	7.2	1130±135	-20±4	<2
MMT/Fe_3O_4_5:1_HC	7.5	625±65	-18±5	<2
MMTK10/Fe_3O_4_1:1_HC	4.6	1120±160	-8±3	<2
MMTK10/Fe_3O_4_5:1_HC	.4.9	330±45	-15±5	<2
LDH2/Fe_3O_4_1:1_HC	8.3	1180±65	2±1	8.4

LDH2/Fe_3O_4_5:1_HC	8.7	1350±195	8±3	9.3
NaB/Fe_3O_4_1:1_HC	6.3	170±40	-25±4	<2
NaB/Fe_3O_4_5:1_HC	6.7	280±75	-21±5	<2
CaB/Fe_3O_4_1:1_HC	5.2	490±95	-11±5	<2
CaB/Fe_3O_4_5:1_HC	6.4	740±230	-14±6	<2
Sep/Fe_3O_4_5:1_HC	5.2	750±200	-15±4	<2

a= nm, b= mV.

Table S3 Real Fe_3O_4 percentage within HC composite samples. Theoretical percentage of Fe_3O_4: 50% for Clay/Fe_3O_4_1:1 samples and 16% for Clay/Fe_3O_4_5:1 samples.

Sample name	Fe_3O_4 real amount (%)
Fe_3O_4	95
MMT/Fe_3O_4_1:1_HC	49
MMT/Fe_3O_4_5:1_HC	16
MMTK10/Fe_3O_4_1:1_HC	39
MMTK10/Fe_3O_4_5:1_HC	5
LDH2/Fe_3O_4_1:1_HC	53
LDH2/Fe_3O_4_5:1_HC	16
NaB/Fe_3O_4_1:1_HC	39
NaB/Fe_3O_4_5:1_HC	12
CaB/Fe_3O_4_1:1_HC	44
CaB/Fe_3O_4_5:1_HC	21
Sep/Fe_3O_4_5:1_HC	17

Table S4 Specific surface area data by BET analysis for heterocoagulated samples.

Sample name	Specific Surface Area ($m^2\,g^{-1}$)
Fe_3O_4	7
MMT	27
MMTK10	250
LDH2	18
NaB	41
CaB	76
Sep	225
MMT/Fe_3O_4_1:1_HC	37
MMT/Fe_3O_4_5:1_HC	30
MMTK10/Fe_3O_4_1:1_HC	185
MMTK10/Fe_3O_4_5:1_HC	160
LDH2/Fe_3O_4_1:1_HC	45
LDH2/Fe_3O_4_5:1_HC	52
NaB/Fe_3O_4_1:1_HC	50
NaB/Fe_3O_4_5:1_HC	51
CaB/Fe_3O_4_1:1_HC	66
CaB/Fe_3O_4_5:1_HC	97
Sep/Fe_3O_4_5:1_HC	230

Table S5 Colloidal characterisation of coprecipitated samples.

Sample name	pH_{nat}	d_{DLS} ^{a}	ζ ^{b}	pH_{IEP}
Fe_3O_4_CP	6.6	115±30	10±3	7.1
MMT@Fe_3O_4_3:1_CP	7.5	530±8	-29±4	<2

MMTK10@Fe_3O_4_3:1_CP	7.7	450±35	-33±7	<2
LDH2@Fe_3O_4_3:1_CP	7.5	1265±10	20±6	9.3
CaB@Fe_3O_4_3:1_CP	7.7	510±30	-28±4	<2
Sep@Fe_3O_4_3:1_CP	8.0	450±120	-27±5	2.6

a= nm, b= mV.

Table S6 Specific surface area data by BET analysis for coprecipitated samples.

Sample	Specific Surface Area ($m^2 g^{-1}$)
Fe_3O_4_CP	78
MMT@Fe_3O_4_3:1_CP	45
MMTK10@Fe_3O_4_3:1_CP	156
LDH2@Fe_3O_4_3:1_CP	66
CaB@Fe_3O_4_3:1_CP	117
Sep@Fe_3O_4_3:1_CP	172

a= $m^2 g^{-1}$.

Table S7 Results of absorption test performed on heterocoagulated samples ($mg_{M+}\ g_{sample}^{-1}$). Relative standard deviations are between 0.01 and 0.06 $mg_{M+}\ g_{sample}^{-1}$.

Sample name	Cu^{2+} adsorption 1h	Cu^{2+} adsorption 24h	Fe^{3+} adsorption 1h	Fe^{3+} adsorption 24h
Fe_3O_4	1.9	2.1	12.2	12.5
MMT	8.9	9.3	15.2	15.3
MMTK10	1.8	2.0	12.4	12.8
LDH2	2.0	2.1	15.5	15.8
NaB	4.8	5.1	14.9	15.6
CaB	8.7	9.0	15.1	15.9
Sep	8.4	8.6	11.5	12.3
MMT/Fe_3O_4_1:1_HC	7.7	7.9	14.5	15.3
MMT/Fe_3O_4_5:1_HC	8.3	8.6	14.9	15.5
MMTK10/Fe_3O_4_1:1_HC	2.4	2.7	13.5	14.2
MMTK10/Fe_3O_4_5:1_HC	2.6	3.0	13.4	14.5
LDH2/Fe_3O_4_1:1_HC	0.1	2.2	15.4	15.6
LDH2/Fe_3O_4_5:1_HC	2.0	2.3	15.5	15.9
NaB/Fe_3O_4_1:1_HC	3.6	3.8	14.4	14.9
NaB/Fe_3O_4_5:1_HC	4.5	4.8	14.4	15.1
CaB/Fe_3O_4_1:1_HC	6.2	6.5	14.8	15.1
CaB/Fe_3O_4_5:1_HC	6.1	6.7	15.1	15.5
Sep/Fe_3O_4_5:1_HC	7.5	7.9	11.9	12.6

Table S8 Results of adsorption test performed on coprecipitated samples ($mg_{M+}\ g_{sample}^{-1}$). Relative standard deviations are between 0.01 and 0.06 $mg_{M+}\ g_{sample}^{-1}$.

Sample name	Cu^{2+} adsorption 1h	Cu^{2+} adsorption 24h	Fe^{3+} adsorption 1h	Fe^{3+} adsorption 24h
Fe_3O_4_CP	0.05	0.1	13.4	13.7
MMT@Fe_3O_4_3:1_CP	2.1	2.4	12.9	13.4
MMTK10@Fe_3O_4_3:1_CP	0.7	0.9	14.1	14.5
LDH2@Fe_3O_4_3:1_CP	0.9	1.0	14.6	15.1

CaB@Fe_3O_4_3:1_CP	2.9	3.1	14.1	14.8
Sep@Fe_3O_4_3:1_CP	3.0	3.3	14.4	14.9

Table S9 Results of dye adsorption by heterocoagulated samples (mg_{dye} g^{-1}). Relative standard deviations are in the range of 1-3%, attributable to the uncertainty associated with the measurement instrument used.

Sample name	RhB adsorption 1h	MO adsorption 1h	MB adsorption 1h
Fe_3O_4	3.5	1.0	0.5
MMT	18.7	2.8	6.4
MMTK10	17.8	0.6	7.0
LDH2	3.4	6.3	0.3
NaB	16.5	0.9	6.8
CaB	12.5	1.2	6.9
Sep	18.5	1.0	7.0
MMT/Fe_3O_4_1:1_HC	18.9	0.7	6.9
MMT/Fe_3O_4_5:1_HC	18.9	0.5	6.7
MMTK10/Fe_3O_4_1:1_HC	18.9	1.2	7.0
MMTK10/Fe_3O_4_5:1_HC	19.7	1.2	7.0
LDH2/Fe_3O_4_1:1_HC	5.1	6.6	0.9
LDH2/Fe_3O_4_5:1_HC	7.2	6.6	0.4
NaB/Fe_3O_4_1:1_HC	13.2	1.2	6.7
NaB/Fe_3O_4_5:1_HC	15.2	0.9	6.7
CaB/Fe_3O_4_1:1_HC	14.0	1.3	7.0
CaB/Fe_3O_4_5:1_HC	18.7	1.0	7.0
Sep/Fe_3O_4_5:1_HC	18.9	1.4	7.0

Table S10 Results of dye adsorption by coprecipitated samples (mg_{dye} g^{-1}). Relative standard deviations are in the range of 1-3%, attributable to the uncertainty associated with the measurement instrument used.

Sample name	RhB adsorption 1h	MO adsorption 1h	MB adsorption 1h
Fe_3O_4_CP	2.4	0.9	1.6
MMT@Fe_3O_4_3:1_CP	10.5	0.6	6.8
MMTK10@Fe_3O_4_3:1_CP	11.3	0.8	7.0
LDH2@Fe_3O_4_3:1_CP	9.6	5.3	3.0
CaB@Fe_3O_4_3:1_CP	7.4	1.1	6.7
Sep@Fe_3O_4_3:1_CP	12.3	0.3	7.0

Table S11 Kinetic parameters for both heterocoagulated and coprecipitated samples against RhB after 1h of adsorption test.

Sample name		Pseudo-First Order Reaction			Pseudo-Second Order Reaction		
	q_e exp.[a]	K_1 [b]	q_e calc. [a]	R_1^2	K_2 [c]	q_e calc. [a]	R_2^2
Fe_3O_4	1.2	1.2*10^-3	10.1	0.91	2.2*10^-2	1.3	0.99
MMT	6.5	9.8*10^-4	3.7	0.97	4.2*10^-2	6.6	0.99
MMTK10	6.2	1.1*10^-3	3.2	0.95	4.7*10^-2	6.3	0.99
LDH2	1.2	7.5*10^-4	0.4	0.86	9.6*10^-2	1.2	0.99
NaB	5.8	9.8*10^-4	0.2	0.95	1.5*10^-1	5.5	1
CaB	4.4	1.1*10^-3	19.1	0.91	1.9*10^-2	4.1	0.99

Sep	6.5	1.4*10^-3	3.9	0.97	1.6*10^-1	6.5	1
MMT/Fe_3O_4_1:1_HC	6.4	2.2*10^-3	33.8	0.87	3.1*10^-2	6.7	0.99
MMT/Fe_3O_4_5:1_HC	6.6	1.5*10^-3	4.1	0.86	5.4*10^-2	6.8	0.99
MMTK10/Fe_3O_4_1:1_HC	5.6	5.8*10^-4	0.2	0.98	1.2*10^-1	6.7	1
MMTK10/Fe_3O_4_5:1_HC	5.5	1.5*10^-4	0.1	0.89	1.2*10^-1	6.9	1
LDH2/Fe_3O_4_1:1_HC	1.8	1.1*10^-3	14.2	0.95	2.1*10^-2	1.9	0.99
LDH2/Fe_3O_4_5:1_HC	2.5	1.8*10^-3	116.4	0.97	9.7*10^-3	2.8	0.98
NaB/Fe_3O_4_1:1_HC	4.6	4.3*10^-4	1.6	0.88	4.3*10^-2	4.6	0.99
NaB/Fe_3O_4_5:1_HC	5.3	8.5*10^-4	2.0	0.94	5.2*10^-2	5.3	0.99
CaB/Fe_3O_4_1:1_HC	4.9	1.1*10^-3	39.8	0.97	1.5*10^-2	5.0	0.99
CaB/Fe_3O_4_5:1_HC	6.6	1.1*10^-3	5.3	0.94	4.1*10^-2	6.7	0.99
Sep/Fe_3O_4_5:1_HC	6.6	7.2*10^-4	0.5	0.97	8.4*10^-2	6.6	1
Fe_3O_4_CP	0.8	7.5*10^-5	1.7	0.99	4.1*10^-2	0.9	0.99
MMT@Fe_3O_4_3:1_CP	3.7	1.2*10^-3	39.8	0.97	1.4*10^-2	3.9	0.99
MMTK10@Fe_3O_4_3:1_CP	3.9	3.2*10^-4	39.8	0.87	6.8*10^-3	4.0	0.98
LDH2@Fe_3O_4_3:1_CP	3.4	8.1*10^-4	199.5	0.92	3.5*10^-3	3.7	0.93
CaB@Fe_3O_4_3:1_CP	2.6	7.4*10^-4	0.3	0.98	1.1*10^-1	2.6	0.99
Sep@Fe_3O_4_3:1_CP	4.3	1.3*10^-3	2.5	0.88	5.9*10^-2	4.3	0.99

a= mg g^{-1}, b= min^{-1}, c= g^{-1} mg^{-1} $min^{-0.5}$.

Table S12 Kinetic parameters for both heterocoagulated and coprecipitated samples against MO after 1h of adsorption test.

**Sample name**		**Pseudo-First Order Reaction**			**Pseudo-Second Order Reaction**		
	q_e exp.[a]	K_1 [b]	q_e calc. [a]	R_1^2	K_2 [c]	q_e calc. [a]	R_2^2
Fe_3O_4	3.0	3.9*10^-4	1.2	0.96	1.3*10^-3	1.1	0.98
MMT	8.9	5.6*10^-4	0.2	0.97	2.2*10^-3	9.1	0.99
MMTK10	1.8	5.2*10^-4	2.0	0.96	3.3*10^-2	1.8	0.99
LDH2	19.8	5.5*10^-4	10.5	0.96	1.7*10^-2	20.1	0.99
NaB	2.9	5.4*10^-4	0.7	0.96	2.4*10^-2	2.9	0.99
CaB	3.7	5.3*10^-4	0.4	0.95	1.1*10^-2	2.9	0.99
Sep	3.2	4.8*10^-4	0.3	0.93	3.6*10^-3	2.6	0.94
MMT/Fe_3O_4_1:1_HC	2.3	6.3*10^-4	1.2	0.99	7.7*10^-3	2.4	0.99
MMT/Fe_3O_4_5:1_HC	1.6	5.7*10^-4	2.8	0.96	2.2*10^-2	1.6	0.99
MMTK10/Fe_3O_4_1:1_HC	4.1	5.8*10^-4	0.4	0.97	1.6*10^-2	4.2	0.99
MMTK10/Fe_3O_4_5:1_HC	3.7	5.3*10^-4	0.4	0.98	1.8*10^-2	3.9	1
LDH2/Fe_3O_4_1:1_HC	20.7	5.3*10^-4	1.2	0.97	2.3*10^-2	20.8	1
LDH2/Fe_3O_4_5:1_HC	20.7	5.4*10^-4	1.2	0.97	4.6*10^-2	20.8	0.99
NaB/Fe_3O_4_1:1_HC	3.8	6.3*10^-4	0.4	0.96	6.9*10^-3	3.9	0.99
NaB/Fe_3O_4_5:1_HC	2.9	5.9*10^-4	0.8	0.96	1.7*10^-2	3.3	0.99
CaB/Fe_3O_4_1:1_HC	3.9	5.5*10^-4	0.4	0.97	1.8*10^-2	4.0	0.99
CaB/Fe_3O_4_5:1_HC	3.3	5.5*10^-4	0.6	0.96	1.8*10^-2	3.3	0.99
Sep/Fe_3O_4_5:1_HC	4.4	5.4*10^-4	0.3	0.96	1.3*10^-2	4.6	0.99
Fe_3O_4_CP	2.9	5.8*10^-4	0.7	0.97	1.4*10^-3	4.4	0.99
MMT@Fe_3O_4_3:1_CP	2.0	6.3*10^-4	1.7	0.96	9.9*10^-3	2.1	0.98
MMTK10@Fe_3O_4_3:1_CP	2.5	5.1*10^-4	0.7	0.92	6.3*10^-3	2.5	0.96
LDH2@Fe_3O_4_3:1_CP	16.5	5.3*10^-4	0.7	0.96	4.5*10^-3	16.7	0.99
CaB@Fe_3O_4_3:1_CP	3.4	5.9*10^-4	0.5	0.97	1.9*10^-2	3.6	0.99
Sep@Fe_3O_4_3:1_CP	0.9	5.3*10^-4	5.4	0.94	1.1*10^-2	1.1	0.98

a= mg g^{-1}, b= min^{-1}, c= g^{-1} mg^{-1} $min^{-0.5}$.

Table S13 Kinetic parameters for both heterocoagulated and coprecipitated samples against MB after 1h of adsorption test.

Sample name	Pseudo-First Order Reaction				Pseudo-Second Order Reaction		
	q_e exp.[a]	K_1 [b]	q_e calc. [a]	R_1^2	K_2 [c]	q_e calc. [a]	R_2^2
Fe_3O_4	0.5	2.8*10^-4	21.9	0.95	6.2*10^-2	0.6	0.95
MMT	6.7	5.3*10^-4	0.1	0.96	2.8*10^-2	6.7	1
MMTK10	7.4	5.3*10^-4	0.1	0.96	9.3*10^-1	7.4	1
LDH2	0.4	5.3*10^-4	63.1	0.95	4.2*10^-2	0.4	0.97
NaB	7.1	5.5*10^-4	0.1	0.96	1.9*10^-1	7.1	1
CaB	7.2	5.3*10^-4	0.1	0.96	3.8*10^-1	7.3	1
Sep	7.4	5.3*10^-4	0.1	0.96	3.7*10^-1	7.4	1
MMT/Fe_3O_4_1:1_HC	7.3	5.3*10^-4	0.1	0.96	1.7*10^-1	7.2	1
MMT/Fe_3O_4_5:1_HC	7.1	5.3*10^-4	0.1	0.97	8.4*10^-2	7.0	1
MMTK10/Fe_3O_4_1:1_HC	7.4	5.3*10^-4	0.1	0.96	1.85	7.4	1
MMTK10/Fe_3O_4_5:1_HC	7.4	5.3*10^-4	0.1	0.96	9.2*10^-2	7.4	1
LDH2/Fe_3O_4_1:1_HC	0.9	5.3*10^-4	7.4	0.96	1.9*10^-2	1.1	0.97
LDH2/Fe_3O_4_5:1_HC	0.4	5.8*10^-4	50.1	0.97	7.5*10^-2	0.4	0.99
NaB/Fe_3O_4_1:1_HC	7.1	5.4*10^-4	0.1	0.97	1.2*10^-1	7.1	1
NaB/Fe_3O_4_5:1_HC	7.1	5.4*10^-4	0.1	0.97	1.3*10^-1	7.1	1
CaB/Fe_3O_4_1:1_HC	7.4	5.4*10^-4	0.1	0.96	6.2*10^-1	7.4	1
CaB/Fe_3O_4_5:1_HC	7.4	5.5*10^-4	0.1	0.97	1.6*10^-1	7.3	1
Sep/Fe_3O_4_5:1_HC	7.4	5.3*10^-4	0.1	0.97	3.7	7.4	1
Fe_3O_4_CP	1.7	5.5*10^-4	2.6	0.95	3.6*10^-2	1.7	0.99
MMT@Fe_3O_4_3:1_CP	7.2	5.4*10^-4	0.1	0.97	1.1*10^-1	7.2	1
MMTK10@Fe_3O_4_3:1_CP	7.4	5.4*10^-4	0.1	0.97	9.2*10^-1	7.4	1
LDH2@Fe_3O_4_3:1_CP	3.2	5.5*10^-4	0.6	0.97	4.1*10^-2	3.2	0.99
CaB@Fe_3O_4_3:1_CP	7.1	5.4*10^-4	0.1	0.96	1.4*10^-1	7.1	1
Sep@Fe_3O_4_3:1_CP	7.3	5.4*10^-4	0.1	0.96	4.7*10^-1	7.4	1

a= mg g^{-1}, b= min^{-1}, c= g^{-1} mg^{-1} $min^{-0.5}$.

Table S14 Kinetic studies for phenols removal. Relative standard deviations associated with $q_{e\,(exp)}$ are in the range of 3-5%, attributable to the uncertainty associated with the measurement instrument used.

Sample name	Pseudo-first order				Pseudo-second order				Weber and Morris – Intraparticle diffusion		
	K_1 [a]	q_e [b] (theor)	q_e [b] (exp)	R_1^2	K_2 [c]	q_e (theor)	q_e (exp)	R_2^2	K [d]	C [e]	R^2
CaB/Fe_3O_4_5:1_HC	$4*10^{-1}$	15.4	30.8	0.428	$9*10^{-3}$	30.9	30.8	0.988	0.6	3.7	0.817
LDH2/Fe_3O_4_5:1_HC	$8*10^{-3}$	3.6	44.0	0.009	$5*10^{-1}$	39.8	44.0	0.993	0.3	30.0	0.189

a= h^{-1}, b= µg g^{-1}, c= µg g^{-1} h^{-1}, d= µg g^{-1} $h^{-0.5}$.
